# Insight into mechanisms of pig lncRNA FUT3-AS1 regulating *E*. *coli* F18-bacterial diarrhea

**DOI:** 10.1371/journal.ppat.1010584

**Published:** 2022-06-13

**Authors:** Zhengchang Wu, Hairui Fan, Jian Jin, Song Gao, Ruihua Huang, Shenglong Wu, Wenbin Bao

**Affiliations:** 1 College of Animal Science and Technology, Yangzhou University, Yangzhou, Jiangsu, P. R. China; 2 College of Veterinary Medicine, Yangzhou University, Yangzhou, Jiangsu, P. R. China; 3 Institute of Swine Science, Nanjing Agricultural University, Nanjing, Jiangsu, P. R. China; INSERM U1220, FRANCE

## Abstract

*Escherichia coli* F18 is a common conditional pathogen that is associated with a variety of infections in humans and animals. LncRNAs have emerged as critical players in pathogen infection, but their role in the resistance of the host to bacterial diarrhea remains unknown. Here, we used piglets as animal model and identified an antisense lncRNA termed FUT3-AS1 as a host regulator related to *E*. *coli* F18 infection by RNA sequencing. Downregulation of FUT3-AS1 expression contributed to the enhancement of *E*. *coli* F18 resistance in IPEC-J2 cells. FUT3-AS1 knockdown reduced *FUT3* expression via decreasing the H4K16ac level of *FUT3* promoter. Besides, the FUT3-AS1/miR-212 axis could act as a competing endogenous RNA to regulate *FUT3* expression. Functional analysis demonstrated that target *FUT3* plays a vital role in the resistance of IPEC-J2 cells to *E*. *coli* F18 invasion. A *Fut3*-knockout mice model was established and *Fut3*-knockout mice obviously improved the ability of resistance to bacterial diarrhea. Interestingly, *FUT3* could enhance *E*. *coli* F18 susceptibility by activating glycosphingolipid biosynthesis and toll-like receptor signaling which are related to receptor formation and immune response, respectively. In summary, we have identified a novel biomarker FUT3-AS1 that modulates *E*. *coli* F18 susceptibility via histone H4 modifications or miR-212/*FUT3* axis, which will provide theoretical guidance to develop novel strategies for combating bacterial diarrhea in piglets.

## Author summary

Piglets’ *E*. *coli* F18-induced diarrhea is one of the most common intestinal inflammatory diseases that leads to severe financial damages. However, the molecular mechanism underlying the resistance to *E*. *coli* F18 infection remains unclear. Here, FUT3-AS1 as a novel long non-coding RNA (lncRNA) was identified and could function as a positive regulator of *E*. *coli* F18 susceptibility in IPEC-J2 cells. FUT3-AS1 not only functions as a sponge by competitively binding to miR-212, but also increases H4K16ac level to promote the binding activity of Sp1 to the *FUT3* core promoter, subsequently upregulating the *FUT3* expression and activating glycosphingolipid biosynthesis and TLR signaling. Besides, we found that *FUT3* is a promising new target for combating *E*. *coli* F18 infection. In summary, our findings illustrated a critical role of FUT3-AS1 in regulating *E*. *coli* F18 susceptibility, and will provide valuable candidates for the molecular breeding of resistance to bacterial diarrhea in piglets.

## Introduction

Enterotoxigenic *Escherichia coli* (ETEC) is an important zoonotic bacterial pathogen. ETEC strains are the most frequent bacterial cause of children and travelers’ diarrhea, resulting in considerable morbidity and mortality in developing countries [1]. Moreover, ETEC strains are also important pathogens in post-weaning piglets causing tremendous damage and loss to the pig industry [2,3]. Porcine pathogenic ETEC strains often harbor specific colonization factors, including fimbrial adhesins F4 (K88) and F18 [4]. Thereinto, *E*. *coli* F18 is the major causative pathogens of bacterial diarrhea. At present, the pathogenesis of *E*. *coli* F18 is relatively clear, which uses its fimbria to attach and subsequently adhere to the brush border F18 receptors of the epithelial cells, resulting in production of enterotoxin, causing diarrhea [5]. Therefore, resistance against *E*. *coli* F18 infection relies on individual immunity as well as receptor expression on intestinal epithelial cells. However, the molecular mechanism underlying the resistance to *E*. *coli* F18-bacterial diarrhea is not fully understood.

LncRNAs (long non-coding RNAs) is a class of non-protein coding transcripts with more than 200 nucleotides in length, and the functional roles for most lncRNAs remained elusive [6]. Evidence has indicated the key regulatory roles of lncRNAs in numerous important biological processes, including embryonic development, epigenetic regulation, translational regulation, etc. [7–9]. In addition, studies have shown that lncRNAs are associated with the development of antiviral infections and autoimmune diseases [10–12]. However, compared with human lncRNAs, studies on the livestock and poultry lncRNAs also have made great progress. In recent years, with the rapid development of high-throughput sequencing technology, great progress has been made in the study of lncRNAs of important livestock and poultry, including pig, cow, sheep, and chicken, mainly focusing on high-throughput sequencing screening of important lncRNAs related to muscle growth [13], fat metabolism [14] and mammary gland development [15]. Recently, studies have screened lncRNAs related to bovine viral diarrhea infection [16] and identified 299 differentially expressed lncRNAs associated with porcine reproductive and respiratory syndrome virus infection using RNA sequencing [17]. However, the regulatory mechanisms underlying the functions of lncRNAs in animal infectious diseases associated with bacterial diarrhea remains unclear.

In this study, the weaned piglets were used as animal model and fed with *E*. *coli* F18 strains. These model piglets were divided into *E*. *coli* F18-sensitive and -resistant individuals by a series of verification [18]. On this basis, we used comparative lncRNA sequencing to identify a lncRNA, designated FUT3-AS1, which has a critical function in anti-*E*. *coli* F18 infection. Function and mechanism analysis revealed that FUT3-AS1 acts as a *FUT3*-based glycosphingolipid biosynthesis/TLR signaling regulatory circuit that modulates *E*. *coli* F18 susceptibility via H4K16ac modification or miR-212/*FUT3* axis. In addition, *Fut3*-knockout (*Fut3*^-^/^-^) mice as animal model were used to verify the relationship between *FUT3* gene and bacterial diarrhea. Our results provide novel insights into the mechanism of lncRNA for regulating *E*. *coli* F18 infection in weaned piglets, and will provide valuable candidates for the molecular breeding of resistance to bacterial diarrhea in pigs.

## Material and methods

### Ethics statement

All experiments were approved by the Institutional Animal Care and Use Committee (IACUC) of Yangzhou University (Mouse: SCXK(Su)2017-0007; Pig: SYXK(Su)2012-0029) and were performed according to the Animal Ethics Procedures and Guidelines of the People’s Republic of China. No other specific permissions were required for these experiments.

### Experimental animals

Meishan piglets (Kunshan Conservation Ltd., Suzhou, Jiangsu, China) and Sutai piglets (Sutai Pig Breeding Center, Suzhou, Jiangsu, China) are used as experimental animals. In previous studies, the experimental piglets were challenged with a daily dose of 4.6×10^8^ CFU of *E*. *coli* F18 strain and the differences of susceptibility were assessed by assays, such as *E*. *coli* F18 bacteria counting, histopathological and *in vitro* adherence assays of intestinal porcine epithelial cells [18,19]. Ultimately, we selected six piglets resistant (Meishan, n = 3; Sutai, n = 3) and six sensitive (Meishan, n = 3; Sutai, n = 3) to *E*. *coli* F18 for lncRNA sequencing. All experimental piglets were humanely sacrificed using intravenous injection of pentobarbital sodium as necessary to euthanasia, and then 11 tissues (ie. heart, liver, spleen, lung, kidney, stomach, muscle, thymus, lymph nodes, duodenum and jejunum) were collected, which were stored in liquid nitrogen for later use.

Knockout mice for CRISPR/CAS9-mediated were used. Male wild-type (WT) (C57BL/6J) mice (8–10 weeks old; 20–22 g) were procured from the Animal Center of the Yangzhou University (Yangzhou, Jiangsu, China). *Fut3* (*Sec1*) transgenic knockout (*Fut3*^−^/^−^) mice (backcrossed to a C57BL/6, 8–10 weeks old, 20–22 g) were procured from Cyagen (Guangzhou, China). Mouse genotypes were determined by performing polymerase chain reaction (PCR) analysis of tail DNA, using the following PCR primers: forward primer (F): 5’-AGGTGACAGAAAGATTCAGAGGTAC-3’; reverse primer (R): 5’-GAGTGAGTGTGAGTGTGCTAGAAAC-3’, and mice were bred to produce homozygous (KO, *Fut3*^−^/^−^) and WT (*Fut3*^+^/^+^) offspring for this study. All mice feeding and breeding were carried out at the Animal Experiment Centre of Yangzhou University (Yangzhou, China). Each mouse was placed in individual cages allowed to freely access irradiated food and sterile acidified water in a specific pathogen free facility.

### Cell culture and lipopolysaccharide (LPS) exposure

Intestinal porcine epithelial cells (IPEC-J2) (provided by the University of Pennsylvania, Philadelphia, USA) were maintained in complete culture medium (Dulbecco’s modified Eagle’s medium: F12 medium = 1:1, 10% fetal calf serum; Gibco BRL, Life Technologies, Carlsbad, CA, USA). As the cells reached 80–90% confluence, they were treated with 0.1 μg/mL LPS (Sigma-Aldrich, St. Louis, MO, USA) for 0, 4, 8, and 12 h. Cellular RNA was extracted in LPS-induced cells and used for qRT-PCR analysis.

### *E*. *coli* F18 adherence assay *in vitro*

*E*. *coli* F18ab {107/86 (O139:K12:H1)} and *E*. *coli* F18ac {2134P (O157:H19)} fimbriae standard strains were offered by the veterinary laboratory at the Institute of Microbiology, University of Pennsylvania. Adherence assays *in vitro* of *E*. *coli* F18 fimbriae standard strain to IPEC-J2 cells were performed as described previously [19]. In brief, *E*. *coli* F18 strains were inoculated to LB culture medium and incubated for 12 h at 200 r/min on a rocking platform. 1×10^9^ CFU of *E*. *coli* bacteria were added into a monolayer of about 5×10^5^ IPEC-J2 cells in each well of a 12-well culture plate (Corning, NY, USA) for 3 h at 37°C. After centrifugation for 5 min at 4,000 rpm, the two types of *E*. *coli* culture supernatants were filtered (pore size, 0.22 μm) and used to resuspend the bacteria, which were then washed three times. To examine the adhesion capacity of *E*. *coli*, a relative quantification method of *E*. *coli* fimbriae protein (*PILIN*) [20] and colony counting were used to calculate the number of bacterial adhesion. Besides, scanning electron microscopy (SEM), indirect immunofluorescence (IFA), and gram staining were used for further assessment of the adhesion level of *E*. *coli* F18 to IPEC-J2 cells [21].

### *E*. *coli* F18 challenge of mice

In total 180 *Fut3-*WT (*Fut3*^+^/^+^) mice and 60 *Fut3-*KO (*Fut3*^−^/^−^) mice were used in the experiments. Among them, 120 WT (*Fut3*^+^/^+^) mice were divided into four groups (n = 30/each group), treated with orally different dose of *E*. *coli* F18ac (0, 1.0×10^8^ CFU, 1.0×10^10^ CFU, 1.0×10^13^ CFU) for evaluated the optimal dose of *E*. *coli* F18ac, the death rate was recorded within seven days. Then, 30 WT (*Fut3*^+^/^+^) mice and 30 KO (*Fut3*^−^/^−^) mice were treated with orally *E*. *coli* F18ac at dose of 1.0×10^10^ CFU, the death rate was recorded within seven days. In addition, 30 WT (*Fut3*^+^/^+^) mice and 30 KO (*Fut3*^−^/^−^) mice were treated with orally *E*. *coli* F18ac at dose of 1.0×10^10^ CFU, and at the day 4, all the surviving mice were killed and duodenum tissues were collected for further experiments.

### Histopathology

For histopathological studies, fixed duodenal tissues from *Fut3-*WT and *Fut3*-KO mice with *E*. *coli* F18 challenge were dehydrated in graded alcohol, cleared through xylene for 10 min and embedded in paraffin. The paraffin blocks were then cut 5 μm thick, mounted on Superfrost microscope slides (Thermo Fisher Scientific, Waltham, MA, USA). The sections were stained with hematoxylin (Thermo Fisher Scientific) followed by rinsing in running tap water and then re-stained with eosin Y (Sigma-Aldrich, St. Louis, MO, USA), dehydrated, cleared, slide-mounted and visualized by a light microscope (Nikon, Tokyo, Japan).

### LncRNA sequencing, miRNA sequencing and data analysis

For lncRNA sequencing, briefly, the total RNA from duodenum tissues of Meishan-resistant (MR, n = 3), Meishan-sensitive (MS, n = 3), Sutai-resistant (SR, n = 3), and Sutai-sensitive (SS, n = 3) piglets was isolated using TRIzol (Invitrogen, Carlsbad, CA, USA). Double-stranded cDNA was prepared from all RNA samples and sequenced on an Illumina Hiseq 2500 platform (Illumina Inc. San Diego, CA, USA) at the Novogene Bioinformatics Institute (Beijing, China). For miRNA sequencing, the sequencing library of duodenum tissues of resistant (n = 3) and sensitive (n = 3) piglets was constructed using the Small RNA Sample Pre Kit following the vendor’s instructions (Illumina). The library was sequenced on the Illumina Hiseq2500 platform by Oebiotech Corporation (Shanghai, China) and 50 bp single-end reads were produced. LncRNA transcriptome and miRNA analysis were performed as per the provided instructions, followed by data analysis as described previously [22]. LncRNA sequencing data has been submitted to NCBI′s SRA repository under BioProject IDs: PRJNA476718, PRJNA476720, PRJNA476721, PRJNA476722. miRNA sequencing data has been submitted to NCBI′s SRA repository under BioProject IDs: PRJNA812730.

### iTRAQ proteomics analysis

Six total protein samples from IPEC-J2 cells (siFUT3-AS1, n = 3; Control, n = 3) were labeled using an iTRAQ Reagent 8PLEX Multiplex Kit (AB Sciex Inc, Foster City, CA, USA) as per the provided instructions. After iTRAQ labeling, sample fractionation was performed using ab SCX chromatography column in an HPLC system Waters 600E (Thermo Fisher Scientific), followed by liquid chromatography with tandem mass spectrometry and data analysis. Proteins expressions with a fold change>1.2 and a *P-*value<0.05 were considered to be significantly differentially expressed.

### RNA interference and overexpression

For lncRNA FUT3-AS1, two small interfering RNA (siRNA) vector (siFUT3-AS1-1, siFUT3-AS1-2) and negative control (siNC) (S1 Table) were chemically synthesized by Ribobio (Guangzhou, China). For *FUT3* gene, overexpressing lentiviral vector (pGLV5-*FUT3*) and a negative control (pGLV5-NC) were synthesized by GenePharma in Suzhou (China). For miR-212, the primers of miR-212 mimics, mimics NC, miR-212 inhibitor and inhibitor NC were designed (S2 Table) and these sequences were synthesized by GeneCreate in Wuhan (China). Then, the recombinant vector was transfected into cultured IPEC-J2 cells using Lipofectamine 2000 Transfection Reagent (Invitrogen) according to the manufacturer’s guidelines. IPEC-J2 cells were seeded into six-well plates at densities of 5×10^5^ cells/well. After 24 h, when the cells reached >70% confluency, the medium was replaced with fresh Opti-MEM medium immediately, and the cells were transfected with 5 μg appropriate plasmid or 100 pmol proper siRNA using 10 μL transfection reagent according to the manufacturer’s instructions. At 6 h after transfection, cells were washed with PBS twice, and the medium was changed with growth medium. Following a 48-h transfection period, total RNA and total protein of different treatment groups were extracted. qRT-PCR or western blot was performed to evaluate the expression of FUT3-AS1 or *FUT3* in IPEC-J2 cells. Finally, IPEC-J2 cells with FUT3-AS1 silencing, *FUT3* overexpression and miR-212 mimics, inhibitor were obtained for further verification.

### CRISPR/Cas9-mediated knockout

Four CRISPR/Cas9-sgRNA strings (S3 Table) were designed as PCR cassettes (Life Technologies). These double-stranded sgRNA oligos were ligated into the lentiCRISPR vector pGK1.2, which co-expresses Cas9 and sgRNA in the same vector. IPEC-J2 cells were transfected with these vectors selected for 1 week with 2.5 μg/ml puromycin (Sigma-Aldrich). T7 endonuclease I (T7E1) assays was performed to analyze the gene knockout efficiency of these four single guide RNAs (sgRNAs). The sgRNAs were amplified with a product size of 581 bp and used the following primers: forward: 5’-CAGGACTCGTGAAGATTGACCAT-3’ and reverse: 5’-TCAGGTTGAAGTACCCGTCCA-3’. Then, the monoclonal population of the stably infected cells was selected by a limiting dilution assay using the green fluorescent protein marker. The knockout cell line was finally established after being verified by western blotting and sequencing.

### Quantitative real-time reverse transcription PCR (qRT-PCR)

Total RNAs from pig tissues and cells were extracted using Trizol (Invitrogen). Then, reverse transcription of RNA was conducted using PrimerScript RT Reagent Kit with gDNA Eraser (Takara Biotechnology (Dalian) Co., Ltd, Dalian, China). The qPCR reaction system contained 10 μL SYBR Green Mixture, 0.4 μl of 50× ROX Reference Dye II, 1 μL template of cDNA, 1 μL of each primer, and 6.6 μL deionized water. The thermal conditions were as follows: 95°C for 15 s, 40 cycles of 95°C for 5 s, 60°C for 30 s. The *GAPDH* and *ACTB* genes were set as the internal controls. All primers for the qRT-PCR assays are listed in S4 Table.

### Western blotting

Total proteins were extracted from IPEC-J2 cells using an NE-PER kit (Nuclear and Cytoplasmic Extraction Reagents) from Thermo Fisher Scientific as per the provided protocol. A bicinchoninic acid (BCA) kit from Nanjing Keygen Biotech (China) was used for normalizing protein levels. Transfer of proteins to polyvinylidene fluoride (PVDF) membranes was done, followed by incubation with relevant primary antibodies for detection [FUT3 (1:600, Biological), FUT2 (1:600, bioss, Woburn, MA, USA), B3GALNT1 (1:800, LifeSpan BioSciences), ST3GAL3 (1:800, LifeSpan BioSciences), Histone H4 (1:500, Proteintech, Wuhan, Hubei, China), Acetyl-Histone H4 (1:500, PTM BIO, Hangzhou, Zhejiang, China), GAPDH (1:2,500, abcam, Cambridge, UK), β-actin (1:2,000, abcam)] and then with secondary antibody [goat anti-rabbit IgG conjugated with horseradish peroxidase (1:5,000, Abcam)].

### Northern blotting

Total RNA extracted from duodenum tissues was used for northern blotting assays. A DNA sequence specific to FUT3-AS1 (nt 2693–3635) was cloned into the vector pcDNA4/Myc-His B. A radioactive RNA probe 942 nt length was prepared using [a-^32^P] CTP (Perkin Elmer, Waltham, MA, USA) and the *in vitro* transcription labeling system Riboprobe (Promega, Madison, WI, USA). Probe sequence: Forward: GATAGAGCCCAATTTCTTACTCT; Reverse: CAGCAACCGTTTCTTGAATACCT.

### RNA-FISH

Fluorescence-conjugated lncRNA FUT3-AS1 probes were used for RNA-FISH. RNA-FISH was performed as previously described [23]. Hybridization was carried out using DNA probe sets (Ribobio, Guangzhou, Guangdong, China) according to the protocol of Guangzhou RiboBio Co., Ltd. IPEC-J2 cells were observed with a FV1000 confocal laser microscope (Olympus).

### RNA pull-down and mass spectrometry

Three Fragments (1–1759 nt, 1760–3949 nt, and 3950–5833 nt) of FUT3-AS1 were sub-cloned from the full-length cDNA sequence. FUT3-AS1 fragments were transcribed with Maxiscript T7 kit (Ambion, Austin, TX, USA) and biotinylated with biotin by Pierce RNA 3’ end desthiobiotinylation kit (Thermo Fisher Scientific) according to the manufacturer’s protocol. Five picomoles of biotinylated RNA was incubated with preblocked streptavidin agarose beads (Invitrogen) at 4°C for 5 h, followed by extensive washes to obtain biotinylated RNA-conjugated agarose beads. To extract FUT3-AS1-interacting proteins, the beads were washed and boiled in SDS buffer, and the retrieved protein was analyzed by 20% SDS-PAGE and silver staining (Pierce silver stain kit, Thermo Fisher Scientific). LC-MS/MS analysis was performed over a 66-min run on a mass spectrometer (TripleTOF 5600+).

### RNA immunoprecipitation (RIP)

RNA immunoprecipitation (RIP) experiments were performed with a Magna RIP RNA-binding protein immunoprecipitation kit (Millipore, Billerica, MA, USA) according to the manufacturer’s instructions. Briefly, IPEC-J2 cells were treated with 1% formaldehyde for crosslinking, and then lyzed with RNase-free RIPA buffer supplemented. The antibodies used for RIP were 5 μg histone H4 (Proteintech), 5 μg Ago2 (Abcam), and 5 μg IgG (Dia-An Biotech, Wuhan, Hubei, China). RNA/protein complexes were immunoprecipitated and RNA was extracted and measured with qRT-PCR. The relative RNA levels were normalized to input and the fold enrichment was calculated as 2^-ΔΔCt^ with normalization to IgG control.

### Chromatin immunoprecipitation (ChIP)

ChIP assays were carried out as previous described protocol [24]. IPEC-J2 cells were subjected to nucleus isolation and sonication and then incubated with anti-histone H4 (16047-1-AP, Proteintech), H4K8ac (PTM-164, PTM BIO), H4K12ac (PTM-165, PTM BIO), H4K16ac (PTM-166, PTM BIO), with protein G magnetic beads (20421, Pierce) together. The DNA was then precipitated and stored at -80°C. The prepared DNA was applied for qPCR using qRT SuperMix II (Vazyme Biotech Co., Ltd., Nanjing, Jiangsu, China) with Real-time quantitative PCR instrument ABI7500 (Applied Biosystems, Foster City, CA, USA) detection system. The enrichment levels were normalized to the input sample.

### Co-immunoprecipitation (CoIP)

For co-immunoprecipitation assays, IPEC-J2 cells were cotransfected with different plasmid combinations as indicated. The purified protein from about 10^8^ cells were lysed with Triton X-100 lysis buffer (40 mM Tris, 120 mM NaCl, 1% Triton X-100, 1 mM NaF, 1 mM Na_3_VO_4_) supplemented with protease inhibitor cocktail. The total protein concentrations of the lysates were measured with BCA assay. Equal amounts of protein were incubated with anti-Flag antibody or IgG on a rotator overnight at 4°C. Then, protein A/G magnetic beads (MCE, Monmouth Junction, NJ, USA) were added and incubated with the lysates for another 4 h. The beads were washed four times with wash buffer followed by the addition of SDS-PAGE loading buffer. Finally, FUT3 and its interacting proteins were purified with the antibody conjugated beads, followed by mass spectrometric analysis or Western blotting.

### DNA pull down

DNA pull-down assay was performed with a DNA pull-down kit (BersinBio, Guangzhou, Guangdong, China) according to the manufacturer’s protocol. Briefly, the sequences of *FUT3* core promoter region (chr.2: g.73171117–g.73171616) were amplified by PCR and tagged with biotin. The biotin-labeled promoter was bound with streptavidin magnetic beads (BeaverBeads Streptavidin, Shanghai Xiyan Scientific Instruments Co., Ltd.) at 4°C for 4 h. The non-biotinylated promoter was used as the negative control. All protein extracted from IPEC-J2 cells in the input group was used as the positive control. The protein-DNA-streptavidin-agarose complex was analyzed with SDS-PAGE.

### His-tag pull-down

The constructed prokaryotic expression recombinant vector pET-28a-FUT3 plasmid was transformed into BL21 (DE3) competent cells (Takara Biomedical Technology). PET-28a-FUT3 bacteria induced by IPTG (Beijing Solarbio Science& Technology Co., Ltd., China) were sonicated at 75% power until the solution was clear and transparent. His-tagged FUT3 protein purification was performed with a His-tag purification nickel column kit (Beijing ComWin Biotech Co., Ltd., China). His pull-down assays were performed after incubating 10 μg His-FUT3 and Ni-NTA agarose (QIAGEN, Hilden, NRW, Germany). The protein was detected by Western blot. The protein complexes were analyzed and identified by LC-MS/MS protein profiling.

### Luciferase reporter assay

The pcDNA3.1-SP1, pGL3-FUT3 and si-FUT3-AS1 plasmids were constructed to validate of association between the transcription factor SP1 and the target gene *FUT3*. Meanwhile, the *FUT3*-WT, *FUT3*-MUT (CAGGCAACCTGGGGCCTGGGC→TGAATGGTTCAAAATTCAAAT), FUT3-AS1-WT, FUT3-AS1-MUT (GGGATGCTGTCTGGAGAAGGCCAAGG→AAAGCATCACTCAAGAGGAATTGGAA) and miR-212 Mimics plasmids were constructed to reveal the mechanism of ceRNA. For different purposes, HEK293T cells in 24-well plates were transfected with relevant recombinant plasmids or control plasmids together with Renilla luciferase reporter. After 48h, the relative light unit of the firefly and Renilla luciferase was recorded using Dual Luciferase Reporter Gene Assay Kit (Beyotime, Shanghai, China) in accordance with the manufacturer’s instructions. Data were normalized by calculating the ratio between firefly luciferase activity and Renilla luciferase activity.

### Statistical analysis

SPSS 18.0 software (IBM Corp., Armonk, NY, USA) was used. Relative quantitative PCR results were examined via the 2^-ΔΔCt^ method. Between groups comparisons were made using Student’s t test. Data represents the mean ± SEM. Differences were significant statistically at *P*<0.05 or *P*<0.01.

## Results

### Pig FUT3-AS1 was identified as a host regulator associated with *E*. *coli* F18 susceptibility according to RNA-seq analysis

To comprehensively identify potential lncRNAs related to *E*. *coli* F18 susceptibility in Meishan and Sutai piglets (a new hybrid between the Meishan and Duroc breeds), we carried out a comparative lncRNA transcriptome analysis of duodenum tissues using RNA sequencing. A total of 870 million raw reads were obtained, with an error rate less than 0.05% (S5 Table). After quality control, approximately 780 million clean reads were obtained by RNA-seq analysis. Nearly 81.18% (75.07–83.46%) of clean reads could be mapped to the reference genome. Among them, about 69.32% (65.22–72.17%) mapped uniquely to the reference genome of pig (S6 Table). The majority of reads were mRNAs (~65%), followed by misc_RNA (S7 Table). To obtain high quality lncRNAs, we identified the potential lncRNAs according to the workflow (S1 Fig). After five filtering steps, 6012 lncRNAs were selected, including 673 antisense lncRNAs, 3624 lincRNAs, 727 intronic lncRNAs, and 988 sense lncRNAs. Based on the further analysis using four methods of CNCI [25], CPC [26], pfam [27] and PhyloCSF [28], we finally obtained 2056 lncRNAs for subsequent analyses. To evaluate the expression levels of lncRNAs and mRNAs, we calculated the FPKM (Fragments Per Kilobase of transcript per Million mapped) read values in each sample. The expression patterns of lncRNAs and mRNAs between the sensitive group (MS, SS) and resistant group (MR, SR) were similar, with only slight differences, while lncRNAs showed a lower expression level than mRNAs. Most of the lncRNAs contained two exons, and were less than 2000 bp in length.

**Fig 1:**
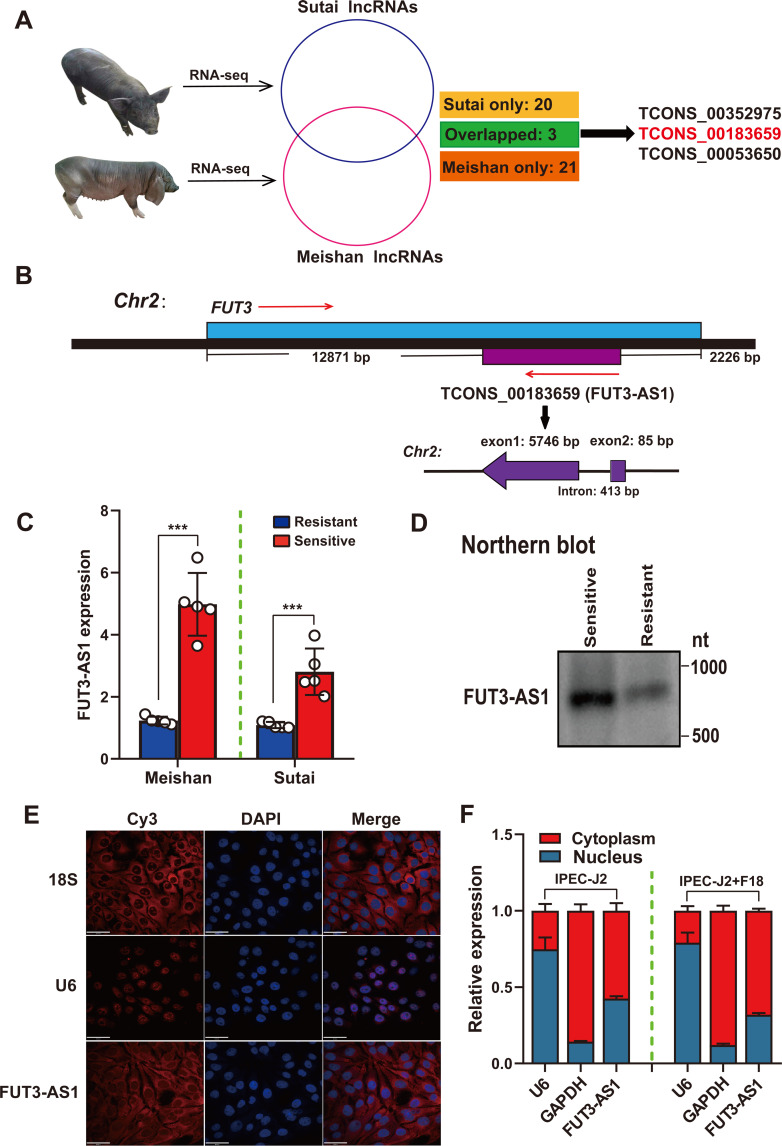
Pig FUT3-AS1 is identified as a host lncRNA involved in *E*. *coli* F18 infection based on RNA-Seq analysis. (**A**) Venn diagram screening of host lncRNAs related to *E*. *coli* F18 infection. The circles represent the differentially expressed lncRNAs between F18-resistant and -sensitive duodenum tissues from Sutai and Meishan piglets, respectively. (**B**) Schematic annotation of the FUT3-AS1 genomic locus on chromosome 2, and the positional relationship between FUT3-AS1 and its target gene. (**C**) qRT-PCR validation of FUT3-AS1 expression in duodenum tissues between F18-resistant and -sensitive piglets (n = 5, means ± SEM; ***P<0.001). (**D**) Northern blotting analysis in duodenum tissues between F18-resistant and -sensitive piglets. (**E**) RNA-fluorescence in situ hybridization (FISH) analysis of FUT3-AS1 in IPEC-J2 cells. DAPI, 4’,6-diamidino-2-phenylindole; Cy3, cyanine 3; *18S* represents a cytoplasmic control, *U6* represents a nuclear control. (**F**) Nuclear-cytoplasmic fractionation assay in normal IPEC-J2 cells and *E*. *coli* F18-infected IPEC-J2 cells. *GAPDH* was considered as a cytoplasmic protein control and *U6* was used as a nuclear control.

To investigate the roles of host lncRNAs in *E*. *coli* F18 infection in weaned piglets, a comparative lncRNA transcriptome analysis was performed in duodenum tissues between F18-resistant and -sensitive piglets from Meishan and Sutai. A total of 24 differentially expressed lncRNAs (|foldchange|>2, *P*-value<0.05) were detected between Meishan F18-resistant and -sensitive piglets group with F18-resistant piglets compared with those in the F18-sensitive group, meanwhile 16 downregulated and 7 upregulated lncRNAs were detected in Sutai F18-resistant piglets compared with sensitive piglets (S2 Fig and S8 and S9 Tables). Interestingly, three lncRNAs were identified in both Sutai and Meishan piglets, such as TCONS_00352975, TCONS_00183659, TCONS_00053650 (Fig 1A). In addition, 46 common differentially expressed genes (DEGs) were obtained between F18-resistant and -sensitive piglets (S3 Fig), including α1,3/4 fucosyltransferase (*FUT3*), which probably correlated with the generation of *E*. *coli* F18 receptor [29,30]. LncRNAs can act in *cis* mechanism to regulate the expression of neighboring genes, we focused on an uncharacterized lncRNA, termed FUT3-AS1 (TCONS_00183659), based on the analysis of *cis* target prediction (S10 Table). FUT3-AS1 is located on chromosome 2 at position 73654921–73661165, overlapping with the antisense strand of the *FUT3* gene (Figs 1B and S4). Furthermore, we verified the differential expression of FUT3-AS1 in duodenum tissues between *E*. *coli* F18-sensitive and -resistant piglets using qRT-PCR (Fig 1C) and northern blotting assay (Fig 1D). To probe the role of FUT3-AS1 in IPEC-J2 cells, we detected the subcellular localization of FUT3-AS1 using RNA-FISH and nuclear-cytoplasmic fractionation. We observed that FUT3-AS1 was present primarily in the nucleus and cytoplasm of normal IPEC-J2 cells or IPEC-J2 cells with *E*. *coli* F18 stimulation (Fig 1E and 1F), which suggested that pig FUT3-AS1 was probably involved in different regulation mechanisms in IPEC-J2 cells. Thus, we preliminarily identified FUT3-AS1 as a host regulator associated with *E*. *coli* F18 susceptibility based on lncRNA transcriptome analysis.

### Downregulation of FUT3-AS1 contributes to the enhancement of *E*. *coli* F18 resistance in IPEC-J2 cells

**Fig 2:**
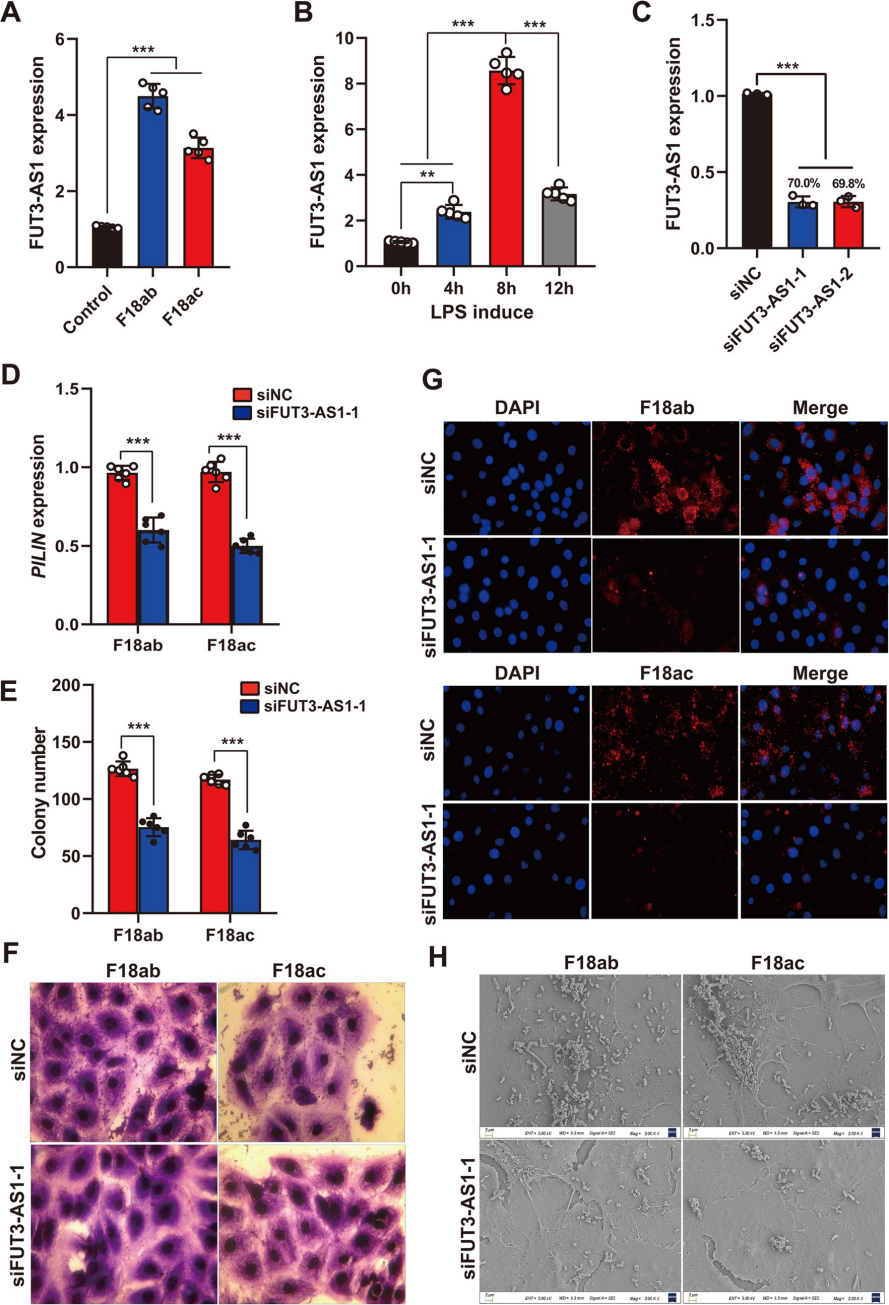
Downregulation of FUT3-AS1 contributes to enhancing *E*. *coli* F18 resistance in IPEC-2 cells. (**A**) Expression changes of FUT3-AS1 in *E*. *coli* F18-stimulated IPEC-J2 cells by using qRT-PCR analysis. IPEC-J2 cells were treated with *E*. *coli* F18ab or *E*. *coli* F18ac strain. (**B**) Expression changes of FUT3-AS1 in LPS-induced IPEC-J2 cells. IPEC-J2 cells were treated with 0.1 μg/mL LPS for 0, 4, 8, and 12 h, n = 5, means ± SEM; **P<0.01, ***P<0.001. (**C**) FUT3-AS1 knockdown in IPEC-J2 cells co-transfected with siFUT3-AS1-1 or siFUT3-AS1-2 vector. The interference efficiency of FUT3-AS1 was assessed by qRT-PCR analysis. (**D**) Expression detection of *E*. *coi* F18 fimbriae gene (*PILIN*) via relative quantification in FUT3-AS1-silenced IPEC-J2 cells. (**E**) Colony number of *E*. *coi* F18 fimbria adhering to IPEC-J2 cells were evaluated, n = 6, mean ± SEM, ***P<0.001. (**F**) Gram staining assay, an optical microscope (400×) was used to observe the IPEC-J2 cells. (**G**) Immunofluorescence assay, blue fluorescence indicates nuclear staining via DAPI; red fluorescence indicates staining with the anti-*E*. *coli* antibody. Cells were observed under a fluorescence microscope (100×). (**H**) Scanning electron microscopy (SEM) assay, cells were observed under a scanning electron microscope (2000×). All of the above *E*. *coi* F18 adhesion experiments were performed in IPEC-J2 cells co-transfected with siFUT3-AS1-1 vector.

To further explore the relationship between FUT3-AS1 expression and *E*. *coli* F18 resistance, we detected the FUT3-AS1 expression in F18ab/ac-stimulated or LPS-induced IPEC-J2 cells. F18-bacterial stimulation and LPS induction both resulted in significantly upregulated expression of FUT3-AS1 in IPEC-J2 cells (Fig 2A and 2B, *P*<0.01). Next, we established FUT3-AS1-silenced IPEC-J2 cells and the knockdown efficiency of FUT3-AS1 expression was determined to be 70.0% in IPEC-J2 cells (Fig 2C). On this basis, we further assessed the effect of FUT3-AS1 knockdown on the level of adhesion of *E*. *coli* F18ab/ac-expressing fimbriae to IPEC-J2 cells (Figs 2D–2H and S5). Relative quantification of *E*. *coli* F18 fimbriae gene (*PILIN*) (Fig 2D) as well as colony counting (Fig 2E) revealed a significantly higher number of bacteria adhering to IPEC-J2 cells in the siFUT3-AS1 group (*P*<0.01). Moreover, the adhesion ability of *E*. *coli* F18 to the IPEC-J2 cells were also examined by gram staining (Fig 2F), indirect immunofluorescence (Fig 2G), scanning electron microscope (Fig 2H). Consistently, the results showed that FUT3-AS1 knockdown decreased the adhesion level of *E*. *coli* F18 to IPEC-J2 cells. Thus, these results clearly suggested the role of FUT3-AS1 in regulating *E*. *coli* F18 infection, and the downregulation of its expression enhances *E*. *coli* F18 resistance in IPEC-J2 cells.

### FUT3-AS1 regulates *FUT3* expression via H4K16ac modification

**Fig 3:**
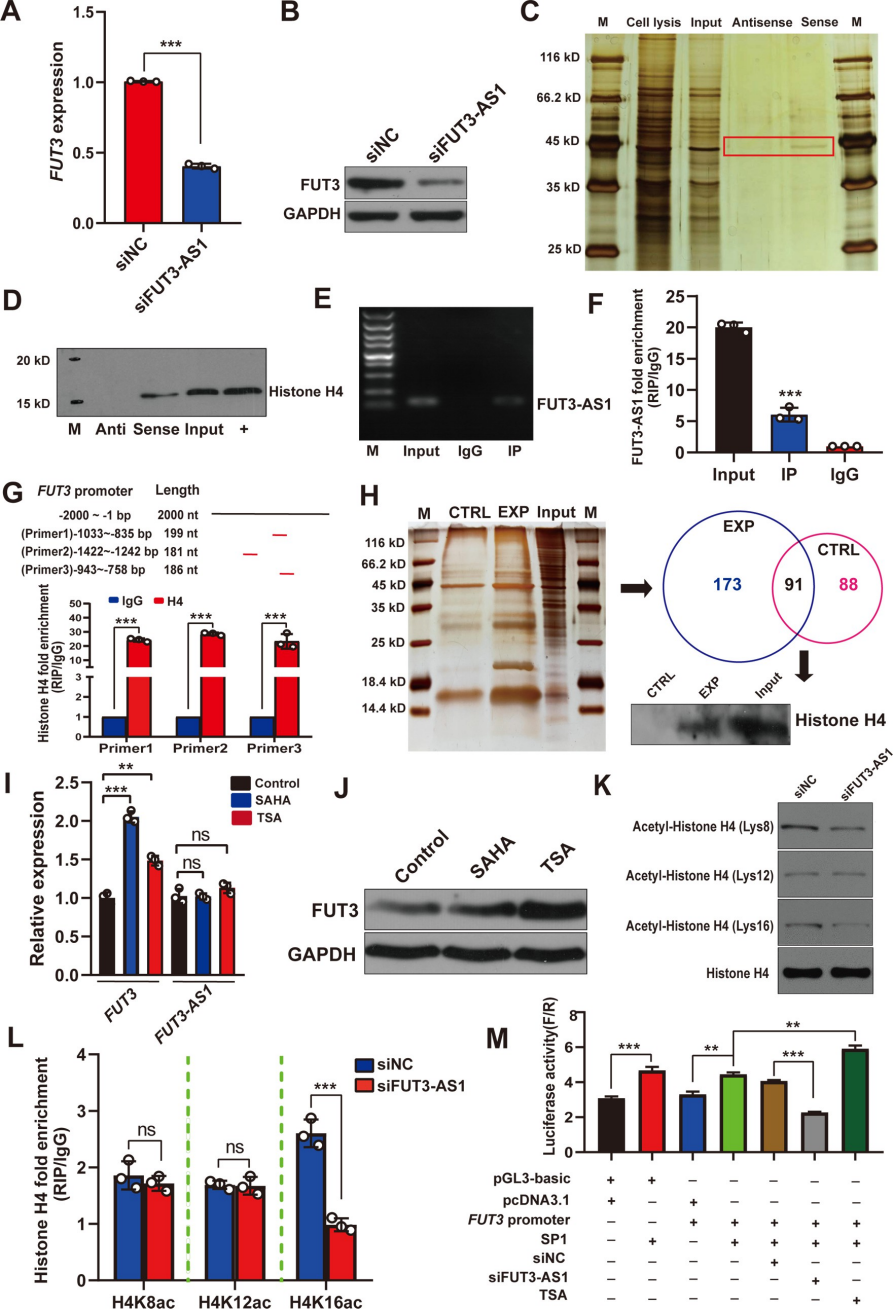
FUT3-AS1 regulates *FUT3* transcription by affecting histone H4K16ac level of the *FUT3* promoter. (**A, B**) *FUT3* expression were detected in IPEC-J2 cells co-transfected with non-silencing hairpin RNA (siNC) and siFUT3-AS1-1 vector using qRT-PCR (A), n = 3, mean ± SEM, ***P<0.001 and western blotting analysis (B), GAPDH as an internal reference. (**C**) RNA pull-down assays of FUT3-AS1-associated proteins in IPEC-J2 cells. FUT3-AS1 fragments corresponding to different regions (1–1759 nt, 1760–3949 nt and 3950–5833 nt) in FUT3-AS1 transcript were labeled by biotin using *in vitro* transcription. Silver staining of biotinylated FUT3-AS1-associated proteins. The FUT3-AS1-specific bands (frame) were excised and analyzed by mass spectrometry, which identified histone H4. (**D**) Western blotting validation of histone H4 from the FUT3-AS1 pull-down assays. (**E, F**) RIP assays of FUT3-AS1 association with histone H4. Nuclear lysates of IPEC-J2 cells were immunoprecipitated with control mouse IgG or anti-histone H4 antibody, and the complexes were analyzed for the presence of FUT3-AS1 using RT-PCR (E) and the enrichment level of FUT3-AS1 was assessed by qRT-PCR (F). (**G**) ChIP assays of histone H4 directly binding to the *FUT3* promoter. Schemes illustrating the 2 kb sequence of the *FUT3* promoter (*Sscrofa11*.*1*, Chr 2:73150510:73152509:1); Three primers mean different binding sites in the *FUT3* promoter. Validation of histone H4 direct binding sites in the *FUT3* promoter by ChIP-qPCR. (**H**) DNA pull-down assays of *FUT3* core promoter (Chr. 2: g.73171117–g.73171616). CTRL: control group; EXP: *FUT3* probe group; Binding proteins were identified by mass spectrometry and western blotting validation of Histone H4 binding to the *FUT3* core promoter. (**I**) qRT-PCR analyzed the expression change of FUT3-AS1 and *FUT3* in IPEC-J2 cells treated with histone deacetylase inhibitors, such as Trichostatin A (TSA) and Vorinostat (SAHA), n = 3, mean ± SEM, **P<0.01, ***P<0.001. (**J**) Western blotting validation of *FUT3* expression in TSA or SAHA treated-IPEC-J2 cells. (**K**) Effect of FUT3-AS1 on the level of histone H4 acetylation in IPEC-J2 cells co-transfected with siNC or siFUT3-AS1-1 vector, as assessed using western blotting, including acetyl-histone H4 (Lys16) (H4K16ac), H4K12ac, H4K8ac. (**L**) Enrichment of acetyl-histone H4 binding to the *FUT3* core promoter was determined using ChIP-qPCR, n = 3, mean ± SEM, ^ns^*P*>0.05, ***P<0.001. (**M**) Effect of FUT3-AS1 on Sp1 binding to the *FUT3* core promoter was measured using a luciferase reporter assay. pGL3-Basic: pGL3 Luciferase Reporter Vectors; *FUT3* promoter: *FUT3* core promoter sequence; siFUT3-AS1: siFUT3-AS1-1 vector.

To identify the target of FUT3-AS1, qRT-PCR and western blotting analysis were used after FUT3-AS1 knockdown, which indicated a significantly reduction in *FUT3* expression (Fig 3A and 3B). Many lncRNAs are reported to function through their interactions with proteins in the nucleus of cells [31]. Therefore, we hypothesized that FUT3-AS1 might affect cellular functions in a similar manner. To probe the underlying molecular mechanism of FUT3-AS1 in IPEC-J2 cells, we performed an RNA pull-down assay to identify proteins that are associated with FUT3-AS1. To cover the whole FUT3-AS1 transcript, we used multiple biotinylated RNA fragments (1–1759 nt, 1760–3949 nt, 3950–5833 nt). RNA-associated proteins were resolved using 10% SDS-PAGE, and the bands specific to FUT3-AS1 were excised and subjected to mass spectrometry (Fig 3C). Mass spectrometry identified 19 FUT3-AS1-associated proteins (S11 Table). Among them, histone H4 was detected by western blotting from RNA pull-down assays (Fig 3D). We further performed RIP assay using an antibody against Histone H4 in IPEC-J2 cells. We observed FUT3-AS1 enrichment using the Histone H4 antibody versus a nonspecific antibody (IgG control) by RT-PCR (Fig 3E) and RIP-qPCR (Fig 3F). To probe the association of target *FUT3* and histone H4, we investigated the binding of histone H4 to the *FUT3* promoter (Sscrofa11.1:2:73150510:73152509:1) using ChIP binding assays. As shown in Fig 3G, histone H4 levels were increased significantly in the promoter regions of *FUT3* compared with the controls (*P*<0.01). Besides, we have identified the core promoter region (chr2: g.73171117–g.73171616) of *FUT3* in previous study [32]. On this basis, we performed a DNA-pull down assay and identified 173 specific *FUT3* promoter-associated proteins using mass spectrometry, including histone H4. Further western blotting validation showed that histone H4 can directly bind to the *FUT3* core promoter (Fig 3H).

To explore which modifications of histone H4 affect *FUT3* gene expression, we detected *FUT3* expression in IPEC-J2 cells treated with histone deacetylase inhibitors, such as trichostatin A (TSA) and vorinostat (SAHA). After TSA or SAHA treatment, *FUT3* showed a significantly upregulated expression (Fig 3I and 3J), whereas there was no significant change in FUT3-AS1 expression (Fig 3I), which indicated that histone acetylation probably regulates *FUT3* expression. Generally, several common modifications of histone H4 acetylation includes acetyl-Histone H4 (Lys16) (H4K16ac), H4K12ac and H4K8ac. Therefore, we assessed the effect of FUT3-AS1 on the level of histone H4 acetylation using western blotting (Fig 3K). Further ChIP-qPCR assays showed that the level of H4K16ac in the *FUT3* core promoter decreased significantly in siFUT3-AS1 cells (Fig 3L). Eukaryotic transcription is a highly regulated process, and histone acetylation plays a crucial role in the binding activity of transcription factors [33]. Using Alibaba (http://www.gene-regulation.com/pub/programs/alibaba2/index.html?) and JASPAR software [34], we identified the transcription factor binding sites (TFBS) in the core promoter region of pig *FUT3*, such as NFIC, NFIX, Sp1, USF, HIF1A, and N-Myc. Dual-luciferase assay showed that NFIC, NFIX, N-Myc, and USF could inhibit the transcriptional activity of the *FUT3* promoter, while Sp1 and HIF1A promoted its transcriptional activity (S6 Fig). The binding of Sp1 to DNA affects transcription-activation and was shown to be associated with *E*. *coli* F18 resistance in weaned piglets [35]. To further explore whether FUT3-AS1 or histone H4 acetylation regulates the binding activity of Sp1 to the *FUT3* core promoter, we also performed a dual-luciferase assay and found that the transcriptional binding activity of Sp1 showed an extremely significant decline in siFUT3-AS1 IPEC-J2 cells, and significantly increased in TSA treated IPEC-J2 cells (Fig 3M). These data clearly suggest that FUT3-AS1 probably promotes *FUT3* expression by increasing the H4K16ac level and Sp1 binding activity on the *FUT3* core promoter.

### FUT3-AS1 binds directly to miR-212 and targets *FUT3*

**Fig 4:**
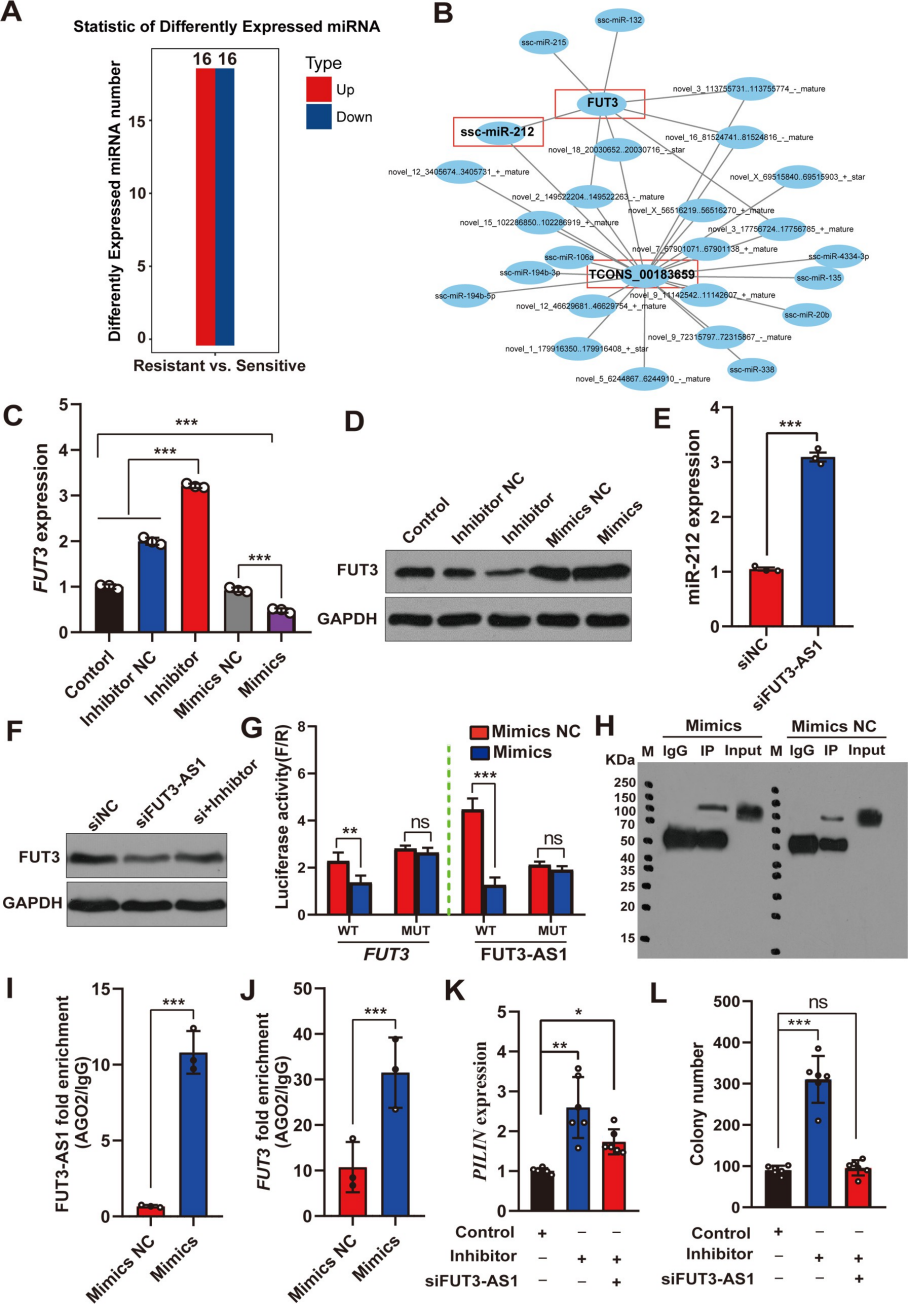
FUT3-AS1 functions as a sponge for miR-212 and targets *FUT3*. (**A**) Analysis of differentially expressed miRNAs in duodenal tissues between F18-resistant and -sensitive piglets by microRNA sequencing. (**B**) ceRNA network construction based on FUT3-AS1 (TCONS_00183659), target *FUT3* and miRNAs. (**C, D**) *FUT3* expression were detected in IPEC-J2 cells co-transfected with miR-212 mimics, inhibitor or miR-NC using qRT-PCR (C) and western blotting (D). (**E**) Detection of miR-212 expression in IPEC-J2 cells co-transfected with siFUT3-AS1-1 or siNC vector. (**F**) Western blotting analysis of *FUT3* expression were detected in IPEC-J2 cells co-transfected with siNC, siFUT3-AS1-1, siFUT3-AS1-1+ miR-212 inhibitor. (**G**) Relative luciferase activities were measured in IPEC-J2 cells co-transfected with miR-212 Mimics or Mimics NC, *FUT3*-WT or *FUT3*-MUT, FUT3-AS1-WT, or FUT3-AS1-MUT. (**H**) RIP experiments were performed using the anti-AGO2 antibody. (**I, J**) Enrichment of *FUT3*, FUT3-AS1 binding to the AGO2 was determined using RIP-qPCR. (**K, L**) The number of *E*. *coi* F18 fimbria adhering to IPEC-J2 cells was assessed by relative quantification (K) and colony counting (L) in different treatment groups including miR-NC (Control), miR-212 inhibitor, miR-212 inhibitor+siFUT3-AS1-1. All data are presented as the mean ± SEM, ^ns^*P*>0.05, *P<0.05, **P<0.01, ***P<0.001.

To further investigate the role of FUT3-AS1 in the cytoplasm of IPEC-J2 cells, we explored whether FUT3-AS1 regulates *FUT3* expression through a miRNA-dependent mechanism. To obtain the potential miRNAs, we initially identified 32 differentially expressed miRNAs associated with *E*. *coli* F18 susceptibility using miRNA sequencing (Fig 4A and S12 Table). Then, we used the bioinformatic tool miRanda v3.3a [36] to further investigate key miRNAs that target FUT3-AS1 and *FUT3* transcripts (S13 Table). ceRNA (competing endogenous RNAs), including long non-coding RNA (lncRNA) and circular RNA (circRNA), could competitively combine to microRNA which will interfere miRNA binding to messenger RNA (mRNA) to regulate gene expression, thereby affecting cell function [37]. According to the prediction, we constructed a ceRNA regulating network that integrated FUT3-AS1 (TCONS_00183659)-miRNAs and miRNAs-*FUT3* (Fig 4B). The results showed that miR-212 has the putative binding sites in FUT3-AS1 and *FUT3* 3’UTR (S7 Fig). Thus, these results suggested that miR-212 might be a critical regulatory miRNA for FUT3-AS1.

To confirm that FUT3-AS1 and *FUT3* are regulated by miR-212, we detected whether miR-212 can affect the levels of target *FUT3*. *FUT3* expression significantly decreased in IPEC-J2 cells in response to miR-212 mimics, whereas *FUT3* increased in response to the miR-212 inhibitor (Fig 4C and 4D). Moreover, we further analyzed the effect of FUT3-AS1 on miR-212 or *FUT3*. Results showed, miR-212 expression was significantly increased in IPEC-J2 cells with siFUT3-AS1 vector (Fig 4E). *FUT3* expression in siFUT3-AS1 cells were slightly reversed when the cells enhanced miR-212 expression (Fig 4F). Using luciferase reporter constructs of mutated (MUT) putative binding sites and wild-type (WT) of *FUT3* transcripts or FUT3-AS1, respectively. As shown in Fig 4G, we observed a markedly lower luciferase activity of the reporter with the WT binding sites of FUT3-AS1 or *FUT3* in IPEC-J2 cells with miR-212 overexpression. However, no variations were observed in luciferase activity for the constructs with mutated FUT3-AS1 binding sites. Later, an RIP assay was performed in IPEC-J2 cells using the antibody against argonaute 2 (Ago2) (Fig 4H). As shown in Fig 4I and 4J, FUT3-AS1 and *FUT3* expression levels were obviously enriched in the Ago2-immunoprecipitate. Taken together, FUT3-AS1 acts as a sponge of miR-212. To further address whether FUT3-AS1 executes its function by interacting with miR-212, we cotransfected miR-212 inhibitor and siFUT3-AS1 expression constructs into IPEC-J2 cells. As shown in Fig 4K, the number of adhesive *E*. *coli* F18 were considerably increased in the miR-212 inhibitor group compared with that in the control groups (*P*<0.01), which was in accordance with the results of *E*. *coi* F18 fimbriae gene (*PILIN*) expression (Fig 4L). However, the effects on *E*. *coli* F18 invasion promoted by miR-212 inhibitor were reversed when the cells reduced FUT3-AS1 expression. In summary, these results demonstrated that FUT3-AS1 probably functions as sponge of miR-212, which increases *FUT3* expression to promote *E*. *coli* F18 susceptibility.

### *FUT3* acts as a potential target for combating *E*. *coli* F18-bacterial diarrhea

**Fig 5:**
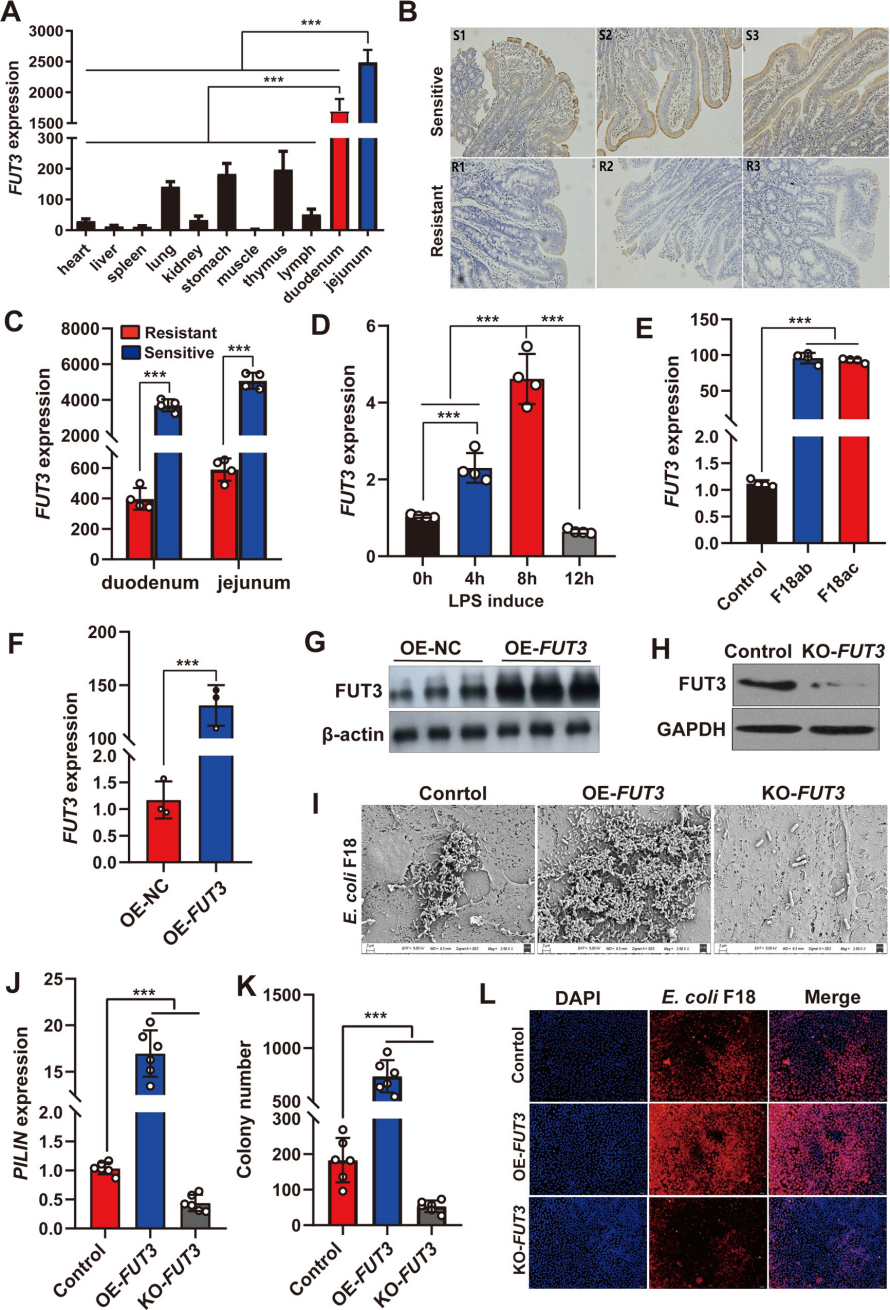
*FUT3* acts as a target to combat *E*. *coli* F18 infection. (**A**) Tissue expression profile of *FUT3* in Meishan weaned piglets at 35 days of age. Eleven tissues including heart, liver, spleen, lung, kidney, stomach, muscle, thymus, lymph, duodenum, jejunum were used for qRT-PCR analysis. (**B**) Immunohistochemical analyses of FUT3 in duodenal tissues between F18-resistant and -sensitive piglets. S: sensitive piglets (n = 3); R: resistant piglets (n = 3) (100× magnification, scale bar = 10 μm). (**C**) Differential expression of *FUT3* in intestinal tissues between resistant and sensitive piglets. (**D, E**) Expression changes of *FUT3* in IPEC-J2 cells after LPS induction (D) and bacteria stimulation (E). IPEC-J2 cells were treated with 0.1 μg/mL LPS for 0, 4, 8, and 12 h; IPEC-J2 cells were treated with *E*. *coli* F18ab or *E*. *coli* F18ac strain. (**F, G**) Overexpression efficiency of *FUT3* gene in IPEC-J2 cells was determined using qRT-PCR (F) and western blotting (G). (**H**) Establishment of *FUT3* knockout (KO-*FUT3*) in IPEC-J2 cells was assessed using western blotting. (**I-L**) Effect of *FUT3* expression on the number of *E*. *coi* F18 fimbria adhering to IPEC-J2 cells was analyzed by scanning electron microscopy (SEM) assay (2500×) (I), relative quantification (J), colony counting (K), immunofluorescence assay (L). IPEC-J2 cells co-transfected with OE-*FUT3*, KO-*FUT3*, or Control. All data are presented as the mean ± SEM, ***P<0.001.

To probe the function of the pig *FUT3* gene, we primarily detected the tissue expression profile of *FUT3* in weaned piglets. As shown in Fig 5A, *FUT3* showed obvious tissue-specific expression, with comparatively high expression in the jejunum and duodenum. Moreover, immunohistochemical analysis showed that FUT3 was mainly distributed in small intestinal epithelial mucosa cells of sensitive piglets (Fig 5B). We further observed that *FUT3* expression was increased significantly in the duodenum and jejunum of *E*. *coli* F18-sensitive compared to *E*. *coli* F18-resistant individuals (Fig 5C). To gain insights into how *FUT3* expression regulated *E*. *coli* F18 invasion, we detected the expression changes of *FUT3* in IPEC-J2 cells after LPS induction (0.1 μg/mL) or *E*. *coli* F18 stimulation. The results of qRT-PCR showed increased the expression of *FUT3* in IPEC-J2 cells induced with LPS (*P*<0.01, Fig 5D) and stimulated with *E*. *coli* F18 strain (*P*<0.01, Fig 5E). Next, we constructed an overexpression vector and designed four sgRNA-cas9 vectors for *FUT3* gene. The level of *FUT3* in IPEC-J2 cells transfected with pcDNA3.1-*FUT3* showed a 150-fold increase (Fig 5F) and the knockout efficiency analysis of *FUT3* was assessed by PCR amplification and sequencing validation (S8 Fig). Further western blotting demonstrated that the protein level of FUT3 showed significant variations in IPEC-J2 cells after overexpression or knockout (Fig 5G and 5H). Besides, we further investigated the effects of *FUT3* expression on the adhesive capacity of *E*. *coli* F18 to IPEC-J2 cells. The adhered-bacteria number of IPEC-J2 were obviously increased in *FUT3* overexpression cells and decreased in *FUT3* knockout cells, as assessed by scanning electron microscopy (Fig 5I), relative quantification (Fig 5J), colony number (Fig 5K) and immunofluorescence assay (Fig 5L). Therefore, these results indicated that *FUT3* probably has a vital role in the susceptibility of IPEC-J2 cells to *E*. *coli* F18 invasion.

**Fig 6:**
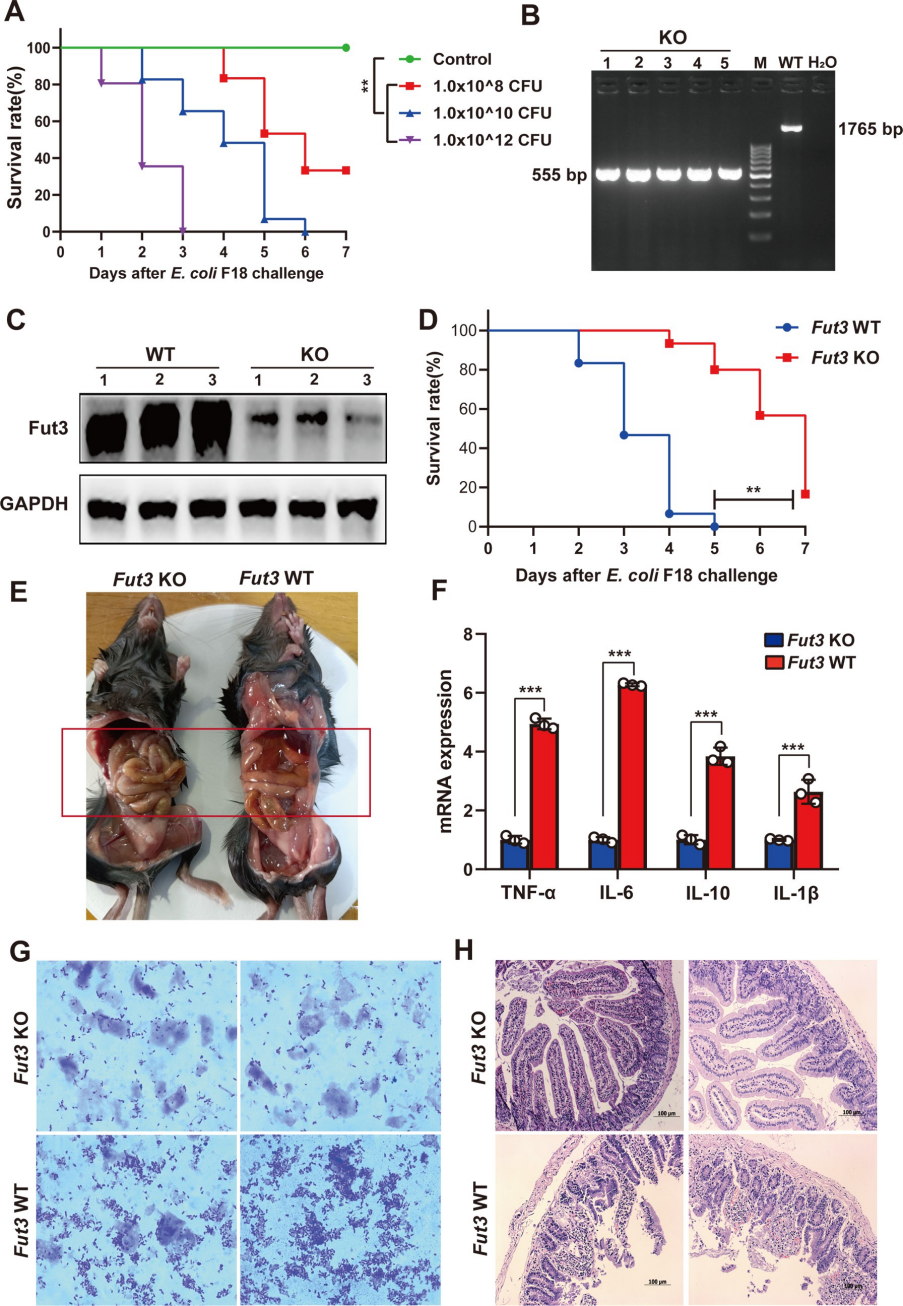
Effect of the *Fut3* gene on *E*. *coli* F18 susceptibility *in vivo*. (**A**) Survival rate analysis of normal mice challenged by *E*. *coli* F18 at different concentrations, including a low concentration group (1 × 10^8^ CFU/mL), a middle concentration group (1 × 10^10^ CFU/mL) and a high concentration group (1 × 10^12^ CFU/mL). The dose was 1 mL/per mouse, n = 30/per group, **P<0.01. (**B**) PCR Screening. *Fut3* knockout (KO) mice: 555 bp; wild type (WT) mice: 1765 bp; M: 100 bp DNA Ladder (Thermo Scientific). (**C**) Western blotting validation of the *Fut3* knockout in mice. (**D**) Survival rate analysis of *Fut3*-KO and *Fut3*-WT mice after 1 × 10^10^ CFU/mL *E*. *coli* F18 challenge. n = 30/per group, **P<0.01. (**E**) Intestinal anatomical diagram of *Fut3*-KO and *Fut3*-WT mice after *E*. *coli* F18 challenge. *Fut3*-KO mice and *Fut3*-WT mice were slaughtered at day 4 after the F18 challenge. (**F**) Partial cytokines analysis of *Fut3*-KO and *Fut3*-WT mice were detected by qRT-PCR, n = 3, mean ± SEM, ***P<0.01. (**G**) Mice brush border bacterial adherence assay. Duodenum brush border cells from *Fut3*-KO and *Fut3*-WT mice were prepared for *in vitro* adherence assay, and the adhesion was observed by using oil-immersion light microscopy. (**H**) Histopathological observation of duodenal tissues in *Fut3*-KO and *Fut3*-WT mice (H&E staining).

We used mice as model animals to further evaluate whether *FUT3* affected *E*. *coli* F18 infection *in vivo*. In normal mice, we determined the optimal dose (1.0×10^10^ CFU/mL) of *E*. *coli* F18 infection using survival rate analysis (Fig 6A). Furthermore, the *Fut3*-knockout (*Fut3*^-^/^-^) mouse model was established by PCR detection (Fig 6B), sequencing (S9 Fig), and western blotting (Fig 6C) analyses. Wild-type (*Fut3*^+^/^+^) and *Fut3*^-^/^-^ mice were challenged with 1.0 × 10^10^ CFU/mL dose of *E*. *coli* F18, and their survival was monitored up to 7 days (Fig 6D). *Fut3*^-^/^-^ mice were less susceptible to *E*. *coli* F18-induced lethality. Intestinal anatomical analysis showed that the intestinal tract of the *Fut3*^+^/^+^ mice appeared obvious pathological reactions, including intestinal hyperemia (Fig 6E). Bacterial diarrhea is an intestinal disease mediated by the overproduction of proinflammatory cytokines, including IL-1β, TNF-α, etc. [38]. We assessed whether *FUT3* controls the release of cytokine in response to *E*. *coli* F18 stimulation *in vivo* by measuring the levels of various cytokines in the *Fut3*^+^/^+^ (WT) and *Fut3*^-^/^-^ (KO) mice after *E*. *coli* F18 challenge (Fig 6F). Compared with the *Fut3* KO mice, the *Fut3* WT mice showed more 2-fold higher levels of IL-6, TNF-α, IL-12, and IL-1β, which indicated that *FUT3* possibly has a crucial role in regulating *E*. *coli* F18-induced cytokine production *in vivo*. Moreover, we further measured the bacteria adhesion level to duodenal mucosa cells in *Fut3* WT and KO mice. As shown in Fig 6G, we found that there was more F18-bacterial adhesion in the *Fut3* WT mice compared with that in the *Fut3* KO mice by gram staining analysis. Histopathological observation showed that *Fut3* WT mice displayed intestinal villi atrophy, exfoliation and inflammatory cells infiltration (Fig 6H). Above results indicated that *Fut3*^-^/^-^ mice showed a better protection against the F18 challenge and the intestinal tract of the *Fut3*^+^/^+^ mice was more prone to lesions. Thus, combined with cell-level results, we speculated that *FUT3* represents a potential new target to combat *E*. *coli* F18 infection in weaned piglets.

### FUT3-AS1 activates FUT3-mediated glycosphingolipid biosynthesis and toll-like receptor (TLR) signaling

**Fig 7:**
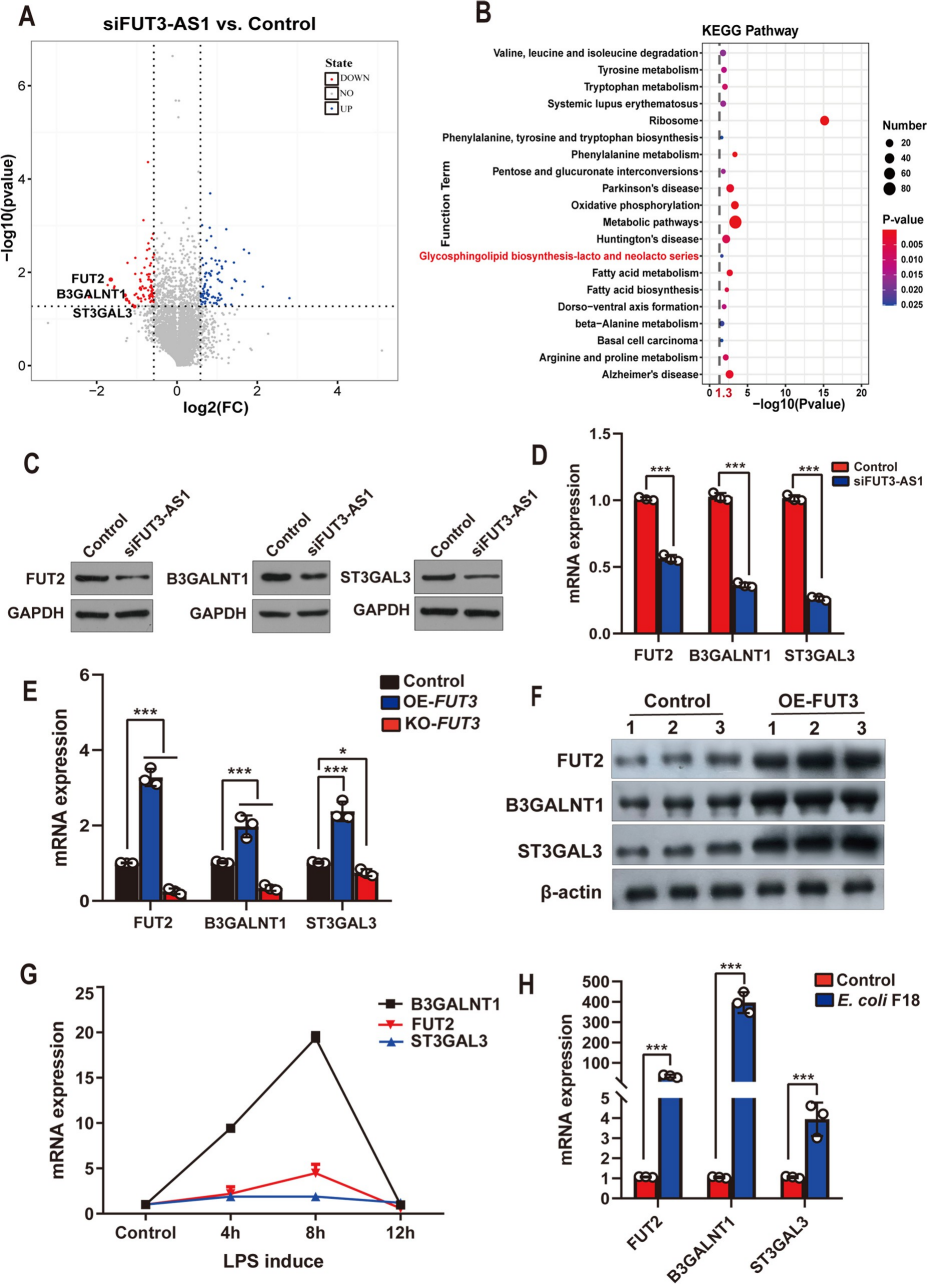
FUT3-AS1 regulates the *FUT3*-mediated glycosphingolipid biosynthesis pathway. (**A**) Volcano plot showing the effect of FUT3-AS1 on the differentially expression proteins (DEPs) in IPEC-J2 cells by iTRAQ proteome analysis. IPEC-J2 cells co-transfected with siNC or siFUT3-AS1-1 vector. The vertical dashed line represents the cutoff for fold change and the horizontal dashed line represents the cutoff for the p-value. Upregulated and downregulated proteins with a p-value less than or equal to 0.05 are denoted by blue and red colored circles, respectively. (**B**) KEGG pathway analysis of differentially expression proteins (DEPs), with the x-axis showing an enrichment factor and the y-axis showing the pathway name; the point size represents the number of DEPs and the point color represents the p-value range. (**C, D**) Expression of glycosphingolipid biosynthesis-associated genes were validated in FUT3-AS1-silenced IPEC-J2 cells and control cells by western blotting (A) and qRT-PCR analysis (B). IPEC-J2 cells co-transfected with siNC or siFUT3-AS1-1 vector. (**E, F**) Effects of *FUT3* on glycosphingolipid biosynthesis-associated genes in IPEC-J2 cells were validated using qRT-PCR (E) and western blotting (F). (**G, H**) Expression of glycosphingolipid biosynthesis-associated genes were detected in LPS-induced (G) and *E*. *coli* F18-stimulated (H) IPEC-J2 cells. All data are presented as the mean ± SEM, *P<0.05, ***P<0.001.

To define the molecular mechanism by which FUT3-AS1 affects *E*. *coli* F18 resistance, we performed a comparative iTRAQ proteome analysis between pig FUT3-AS1 knockdown and control IPEC-J2 cells. FUT3-AS1 knockdown altered the abundance of 388 proteins in IPEC-J2 cells (Fig 7A), including fucosyltransferase 2 (FUT2), beta-1,3-N-acetylgalactosaminyltransferase 1 (B3GALNT1) and alpha2,3-sialyltransferase (ST3GAL3) (S14 Table), which are involved in the pathway of glycosphingolipid biosynthesis (Fig 7B). To verify the effect of FUT3-AS1 on glycosphingolipid biosynthesis, western blotting and qPCR analysis confirmed that the levels of the selected proteins (FUT2, B3GALNT1, and ST3GAL3) from the glycosphingolipid biosynthesis pathway were markedly lower in IPEC-J2 cells after FUT3-AS1 knockdown (Fig 7C and 7D). To explore the role of *FUT3* in glycosphingolipid biosynthesis, we determined the expression of pathway proteins in *FUT3*-knockout or *FUT3*-overexpressing IPEC-J2 cells. After *FUT3* knockout or overexpression, the expression levels of *FUT2*, *ST3GAL3* and *B3GALNT1* changed significantly in IPEC-J2 cells, as assessed using qRT-PCR (Fig 7E) and western blotting analysis (Fig 7F). In addition, we detected the expression changes of glycosphingolipid biosynthesis pathway genes (*FUT2*, *ST3GAL3*, *B3GALNT1*) in LPS-exposed (0.1 μg/mL) IPEC-J2 cells for 4, 8, and 12 h. As shown in (Fig 7G), the levels of *FUT2* and *B3GALNT1* were increased significantly at 4 and 8 h. qRT-PCR analysis showed that *FUT2*, *ST3GAL3*, and *B3GALNT1* expression levels were markedly upregulated in *E*. *coli* strain-stimulated IPEC-J2 cells (Fig 7H). Taken together, these results revealed that FUT3-AS1 positively regulates *FUT3* expression to activate glycosphingolipid biosynthesis signaling.

**Fig 8:**
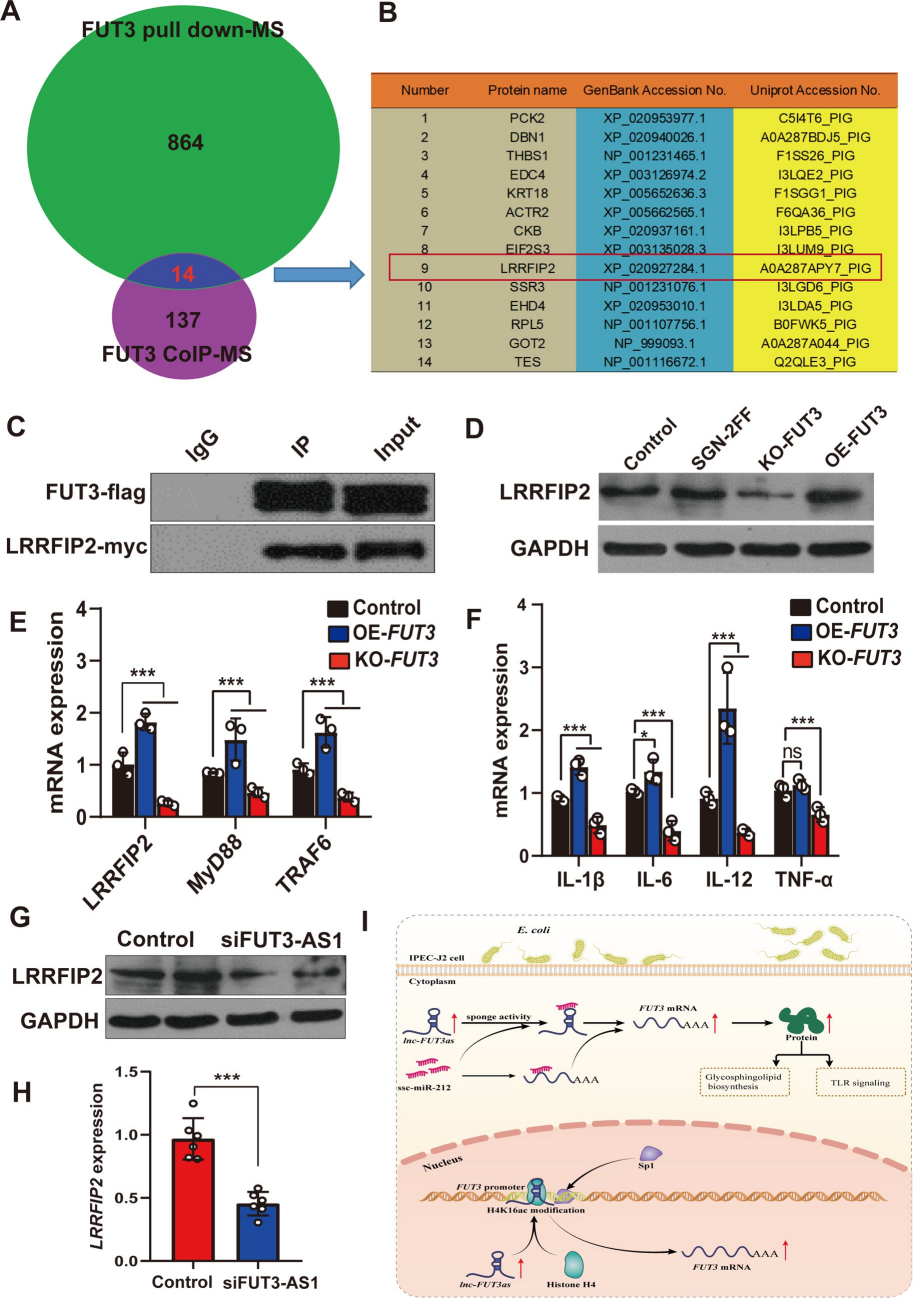
*FUT3* interacts with LRRFIP2 to regulate *TLR* signaling. (**A**) Venn diagram screening of FUT3 interacting proteins by using co-immunoprecipitation (CoIP) and his-tag pull-down and mass spectrometry (MS). (**B**) Detailed information of FUT3 interacting proteins identified by CoIP-MS and pull down-MS. (**C**) Western blotting validation of FUT3’s interaction with LRRFIP2 via CoIP assay. (**D**) Western blotting analysis of LRRFIP2 expression in IPEC-J2 cells co-transfected with *FUT3* overexpression (OE-*FUT3*), *FUT3* knockout (KO-*FUT3*) or fucosyltransferase inhibitor (SGN-2FF) treatment. (**E, F**) Effect of *FUT3* on the expression of pathway-associated genes (E) and partial cytokines (F) in TLR signaling was measured using qRT-PCR. (**G, H**) Effect of FUT3-AS1 on LRRFIP2 expression was measured in FUT3-AS1-silenced IPEC-J2 cells using western blotting (G) and qRT-PCR (H). IPEC-J2 cells co-transfected with siNC or siFUT3-AS1-1 vector. (**I**) Schematic diagram illustrating the mechanism of FUT3-AS1 regulation of *E*. *coli* F18 susceptibility in IPEC-J2 cells. FUT3-AS1 not only functions as a sponge by competitively binding to miR-212, but also increases the H4K16ac level of *FUT3* core promoter, subsequently upregulating the *FUT3* expression to activate glycosphingolipid biosynthesis and TLR signaling. All data are presented as the mean ± SEM, ^ns^*P*>0.05, *P<0.05, ***P<0.001.

To further confirm the FUT3-interacting proteins, we performed a His-pull down assay and CoIP assay of pig FUT3 in IPEC-J2 cells, and then identified the FUT3-interacting proteins using mass spectrometry (MS). Through the combined analysis of CoIP-MS assay (S10 Fig) and pull down-MS assay (S11 Fig), we identified 14 important proteins that interact with FUT3, including LRRFIP2 (Leucine-rich Repeat Fli-I-interacting Protein 2) (Fig 8A and 8B). Further CoIP-western blotting verification showed that FUT3 interacts with LRRFIP2 (Fig 8C). To explore the association between LRRFIP2 expression and fucosyltransferase, we found no significant difference in fucosyltransferase inhibitor (SGN-2FF) treated IPEC-J2 cells, but LRRFIP2 expression showed obvious changes in *FUT3*-knockout or overexpressed IPEC-J2 cells (Fig 8D), which indicated that LRRFIP2 could be regulated by the expression level of *FUT3* rather than its catalytic activity. The role of LRRFIP2 is that of a positive regulator for TLR signaling activation [39]. To verify the effect of *FUT3* on LRRFIP2/TLR signaling, we detected the expression levels of pathway genes (*LRRFIP2*, *MyD88*, *TRAF6*) or proinflammatory cytokines (IL-6, TNF-α, IL-12, IL-1β) in *FUT3* knockout and overexpressed IPEC-J2 cells (Fig 8E and 8F). *FUT3* knockout significantly reduced the levels of *LRRFIP2*, *MyD88*, *TRAF6*, IL-6, TNF-α, IL-12 and IL-1β in TLR4 signaling (*P*<0.01). Conversely, *FUT3* overexpression could enhance its expression. Moreover, western blotting and qPCR analysis confirmed that FUT3-AS1 knockdown also significantly reduced the expression levels of *LRRFIP2* (Fig 8G and 8H). These results indicated that FUT3-AS1 probably triggers *FUT3* expression to activate LRRFIP2/TLR signaling.

In summary, we designed the schematic diagram illustrating the mechanism of FUT3-AS1 regulation of *E*. *coli* F18 susceptibility in IPEC-J2 cells (Fig 8I). The map illustrating mechanisms associated with lncRNA FUT3-AS1 regulating *E*. *coli* F18 susceptibility in IPEC-J2 cells. FUT3-AS1 not only functions as an oncogene by competitively binding to miR-212, but also increases H4K16ac level to promote the binding activity of Sp1 to the *FUT3* core promoter, subsequently upregulating the *FUT3* expression. Meanwhile, lnc-FUT3as acts as a positive regulator of *E*. *coli* F18 susceptibility by activating *FUT3*-mediated glycosphingolipid biosynthesis and TLR signaling.

## Discussion

To date, there has been no report on the molecular mechanism of lncRNAs related with the resistance to *E*. *coli* F18-bacterial diarrhea in piglets. In this study, we used comparative lncRNA sequencing analysis to identify FUT3-AS1 as a key lncRNA in *E*. *coli* F18-sensitive and -resistant duodenal tissues of weaned piglets. FUT3-AS1 was highly expressed in IPEC-J2 cells. IPEC-J2 cells are a relatively new model to study *in vitro* the adherence and pathogenesis of enterotoxigenic *E*. *coli* [40]. FUT3-AS1 expression was upregulated significantly in IPEC-J2 cells post stimulation with *E*. *coli* F18 or induction with LPS. In addition, the adhesion ability of *E*. *coli* F18 to IPEC-J2 cells was reduced significantly by FUT3-AS1 knockdown. These data suggested that FUT3-AS1 plays a critical role in enhancing the resistance of piglets to *E*. *coli* F18; however, its regulatory mechanism remains unclear.

Previous studies demonstrated that lncRNAs have a significant role in gene transcriptional regulation and other cellular processes [41]. In general, the functions of lncRNAs vary according to their different subcellular localizations. Cytoplasmic lncRNAs mainly function as competing endogenous RNAs (ceRNA), whereas the nuclear lncRNAs mainly participate in histone modification and gene transcription regulation [42]. In this study, we demonstrated that FUT3-AS1 was expressed in the nucleus as well as the cytoplasm of IPEC-J2 cells, suggesting FUT3-AS1 affects *E*. *coli* F18 resistance in IPEC-J2 cells through two mechanisms. LncRNAs have a significant role in epigenetic regulation. LncRNAs were discovered to interact with other epigenetic forms and could regulate gene expression by modification of histone methylation/acetylation [8,43]. To date, the studies have focused on the regulation of infectious diseases by lncRNA-mediated histone modifications [11,12,44]. However, there is no relevant report on their involvement in *E*. *coli* diarrhea in piglets. In the present study, the interaction between FUT3-AS1 and histone H4 was confirmed by RNA pull down and RIP assays, and then a further ChIP assay showed that FUT3-AS1 affected the H4K16ac acetylation of the *FUT3* promoter region. Previous studies revealed that chromosome structure, protein interaction, and transcriptional activity were regulated by histone modifications [45]. One of the commonly occurring epigenetic modifications includes Lys16 acetylation in histone H4 (H4K16ac), which is important for a range of fundamental processes, such as DNA damage repair, chromatin decompaction, and transcriptional regulation [46–48]. The results of the present study showed that FUT3-AS1 promoted the H4K16ac acetylation level of the *FUT3* promoter. Histone acetylation is involved in transcriptional activation by modifying the binding activity of transcription factors [33]. We further discovered that FUT3-AS1 could promote the transcriptional binding activity of Sp1 to the *FUT3* promoter using a dual-luciferase assay. Histone acetylation-dependent transcription factors include Sp1 (specificity protein 1), whose binding to DNA activates transcription [49–51]. Thus, our findings suggested that FUT3-AS1 enhances *FUT3* expression via H4K16ac acetylation by activating the binding of Sp1 to the *FUT3* promoter.

Numerous studies have demonstrated that lncRNAs act as ceRNA by binding to and sponging miRNAs in pigs [52,53]. During further exploration of the mechanism of FUT3-AS1 in regulating resistance to *E*. *coli* F18, we found that FUT3-AS1 acted as a ceRNA for miR-212, thus protecting *FUT3* mRNA from degradation to enhance *E*. *coli* F18 susceptibility. Therefore, identifying an miRNA/lncRNA interaction model might facilitate in understanding the underlying mechanism of resistance against *E*. *coli* F18. Thus, according to the above exploration of the two mechanisms of FUT3-AS1, we identified an important target, *FUT3*, to regulate *E*. *coli* F18 susceptibility. A critical step of microbial infection is the attachment of pathogens to host cell glycans. Some microbial pathogens, such as rotaviruses (RVs) and enterotoxigenic *Escherichia coli* (ETEC) causing severe diarrhea in human and animals, also bind to histo-blood group antigens [54,55]. Studies showed, F18-fimbriated *E*. *coli* can selectively interact with glycosphingolipids having histo-blood group antigens (HBGAs) [29,30]. *FUT3* (α1,3/4 fucosyltransferase), belonging to the fucosyltransferase family, participates in regulating the formation of histo-blood group antigens (HBGAs), which include the ABH and Lewis antigens [54]. At present, there are few reports about *FUT3* in pigs. In our previous study, we preliminary found the downregulated expression of *FUT3* was probably associated with *E*. *coli* F18 resistance in IPEC-J2 cells. Additionally, to explore the mechanism of DNA methylation regulating *FUT3* gene expression, we also determined the core promoter (chr.2: g.73171117-g.73171616) of *FUT3* gene, and found 9 methylated CpG sites in the core promoter by bisulfifite amplicon sequencing (BSAS). Further analysis showed the methylation levels of two CpG sites (mC-3, mC-5) located in HIF1A and Sp1 transcription factor have certain inhibitory effect on *FUT3* expression [32]. In this study, our findings revealed that the lncRNA regulated *FUT3* expression via H4K16ac modification or ceRNA mechanism, and systematic experiments demonstrated that *FUT3* acts as a positive regulator of *E*. *coli* F18 susceptibility in *FUT3-*knockout and over-expressed IPEC-J2 cells, meanwhile we further used a *Fut3* knockout mouse model to verify that *FUT3* could affect the body’s resistance against *E*. *coli* F18 diarrhea. Thus, we could confirm *FUT3* as a potentially novel target in pigs for combating *E*. *coli* F18 infection.

The expression levels of specific genes are regulated by different mechanisms involving lncRNAs to activate signaling pathways and affect their biological functions [56–59]. In this study, iTRAQ proteomic and western blotting analyses confirmed the effect of FUT3-AS1 on *FUT3*-mediated glycosphingolipid biosynthesis signaling. Studies showed that glycosphingolipid biosynthesis correlated with the generation of the receptor for *E*. *coli* F18 [29,30]. Combining our findings, we speculated that FUT3-AS1 probably promotes the production of the *E*. *coli* F18 receptor by activating glycosphingolipid biosynthesis signaling. In addition, we identified LRRFIP2 as an important FUT3-interacting protein using CoIP-MS and His pull down-MS. Recently, a class of proteins containing a LRR motif was found to play the pivotal role in recognition of diverse molecules associated with pathogens in host innate defense in animals [59]. LRRFIP2 functions as a positive regulator to activate TLR signaling during the early host response after stimulation with LPS [39]. TLRs can sense diverse pathogenic products that activate host cells’ innate immune/inflammatory response [60], thus playing a vital role in the resistance against *E*. *coli* F18 infection. Therefore, we speculated that FUT3-AS1 also enhances the immune response in IPEC-J2 cells by activating TLR signaling.

In sum, we have identified a lncRNA-based glycosphingolipid biosynthesis/toll-like receptor signaling regulatory molecule that modulates *E*. *coli* F18 susceptibility in piglets. FUT3-AS1 regulates the expression of target *FUT3* by affecting the H4K16ac level of *FUT3* promoter and acting as a sponge for miR-212. Resistance to *E*. *coli* F18 depends on the receptor expression and individual immunity on the pig intestinal epithelium. Studies demonstrated the F18 adhesin receptor is associated with ABH glycosphingolipid binding [28,29]. For *FUT1* gene, a polymorphism (G/A^M307^) significantly reduced fucosyltransferase activity to control the expression of *E*. *coli* F18 receptor [61]. *FUT1* M307 genetic marker, although suitable for foreign pig breeds, but its polymorphism distribution in more than 20 Chinese local pig breeds and wild boar population is extremely skewed [62,63]. The well-known α-1,2-fucosyltransferase encoded by the *FUT2* gene is responsible for the presence of ABH blood-group antigens [54]. However, our findings indicated that *FUT3* not only regulates the formation of F18 receptor by participating in ABH glycosphingolipid biosynthesis, but also improves intestinal immunity by activating TLR signaling in pigs. Thus, our results for the first time provide a new insight into the mechanism of lncRNA regulating the anti-*E*. *coli* F18 infection and confirmed a valuable candidate in weaned piglets, and provided theoretical guidance for solving the problem of molecular breeding for diarrhea disease resistance in pigs.

## Supporting information

S1 Data Excel spreadsheet containing, in separate sheets, the underlying numerical data and statistical analysis for Fig panels 1C, 1F, 2A, 2B, 2C, 2D, 2E, 3A, 3F, 3G, 3I, 3L, 3M, 4C, 4E, 4G, 4I, 4J, 4K, 4L, 5A, 5C, 5D, 5E, 5F, 5J, 5K, 6A, 6D, 6F, 7D, 7E, 7G, 7H, 8E, 8F and 8H. figshare. Dataset. https://doi.org/10.6084/m9.figshare.19656735.v2.
(XLSX)

S1 Fig Comprehensive evaluation and screening of potential lncRNAs. (**A**) The screening workflow for potential lncRNAs. (**B**) lncRNA classifications. ‘x’, ‘u’, ‘i’, and ‘o’ represent antisense lncRNA, intergenic lncRNA (lincRNA), intronic lncRNA, and sense lncRNA, respectively. ‘ = ‘ for complete match of intron chain, ‘c’ for contained, ‘j’ for potentially novel isoform, ‘s’ for an intron of the transfrag overlaps a reference intron on the opposite strand. (**C**) Coding potential analysis of lncRNAs based on four computational approaches (CNCI, CPC, PFAM, and PhyloCSF). (**D**) The FPKM distribution is shown in a box plot, showing no significant differences among the different groups. (**E**) Expression levels of lncRNAs and mRNAs are shown as a violin plot, showing certain differences between lncRNAs and mRNAs. (**F**) Structural comparison between lncRNAs and mRNAs in terms of exon number. (**G**) LncRNAs and mRNAs transcript length comparison.
(TIF)

S2 Fig Volcano plot analysis of differential expression lncRNAs between F18-resistant and -sensitive piglets. (**A**) Volcano plot analysis of differential expression lncRNAs between Meishan F18-resistant and -sensitive piglets. (**B**) Volcano plot analysis of differential expression lncRNAs between Sutai F18-resistant and -sensitive piglets.
(TIF)

S3 Fig Volcano plot analysis of differential expression mRNAs between F18-resistant and -sensitive piglets. **(A**) Volcano plot analysis of differential expression mRNAs between Meishan F18-resistant and -sensitive piglets. (**B**) Volcano plot analysis of differential expression mRNAs between Sutai F18-resistant and -sensitive piglets. (**C**) Venn diagram screening of host mRNAs related to *E*. *coli* F18 infection. (**D**) Common differentially expressed genes (DEGs) between *E*. *coli* F18-resistant and sensitive individuals from Meishan and Sutai piglets.
(TIF)

S4 Fig Coverage tracks analysis of lncRNA FUT3-AS1 from RNA-seq data between F18-resistant and -sensitive individuals. MS represents Meishan F18-sensitive piglets (n = 3); MR represents Meishan F18-resistant piglets (n = 3); SS represents Sutai F18-sensitive piglets (n = 3); SR represents Sutai F18-resistant piglets (n = 3).
(TIF)

S5 Fig Effect of FUT3-AS1 expression on *E*. *coli* F18 adhesion in IPEC-2 cells. (**A**) Expression detection of *E*. *coi* F18 fimbriae gene (*PILIN*) via relative quantification in FUT3-AS1-silenced IPEC-2 cells. (**B**) Colony number of *E*. *coi* F18 fimbria adhering to IPEC-J2 cells were evaluated, n = 6, mean ± SEM, ***P<0.001. (**C**) Immunofluorescence assay, blue fluorescence indicates nuclear staining via DAPI; red fluorescence indicates staining with the anti-*E*. *coli* antibody. Cells were observed under a fluorescence microscope (100×). (**D**) Gram staining assay, an optical microscope (400×) was used to observe the cells. (**E**) Scanning electron microscopy (SEM) assay, cells were observed under a scanning electron microscope (4000×).
(TIF)

S6 Fig Dual-luciferase assay of transcription factor. Transcriptional activity determination of NFIC, NFIX, SP1, USF, HIF1A, and N-Myc in the *FUT3* core promoter using a dual-luciferase assay. The obtained results are indicated as the mean ± SEM, **P<0.01.
(TIF)

S7 Fig Schematic diagram of the binding sites for FUT3-AS1, miR-212, and *FUT3*. (TIF)

S8 Fig Knockout efficiency analysis of the *FUT3* gene. (**A**) PCR results of knockout vector transfected cells, where Blank represents blank cells, and KO-1~4 are cells transfected with different knockout vectors. (**B**) Digestion results of PCR products of knockout vector transfected cells. (**C**) PCR sequencing results of knockout vector-transfected cells. (**D**) knockout single cell PCR results, where 1 represents blank cells, 2~6 represents the knockout single cells. (**E**) PCR sequencing results of the transfected cells with the knockout vector; the last line represents blank untreated cells.
(TIF)

S9 Fig Sequencing confirmation of *Fut3* knockout (KO) mice. *Fut3* deletion fragment (1210 bp) was identified by sequencing.
(TIF)

S10 Fig Identification of *FUT3-*interacting proteins by CoIP-Mass spectrometry (MS). (**a**) Validation of the expression of the FUT3-flag protein by western blotting. (**b**) CoIP assay results. (**c**) Numbers of proteins identified using mass spectrometry.
(TIF)

S11 Fig Identification of FUT3-interacting proteins using His pull down-Mass spectrometry. (**a**) His-pull down assay results. (**b**) Numbers of proteins identified using mass spectrometry.
(TIF)

S1 Table Primer sequences of pig FUT3-AS1 siRNAs. (DOCX)

S2 Table Primer sequences of miR-212 mimics and inhibitor. (DOCX)

S3 Table Single-strand DNA sequences for pig *FUT3* knockout. (DOCX)

S4 Table Real-time PCR primers and sequences. (DOCX)

S5 Table Sequencing data quality summary. (DOCX)

S6 Table Summary of Illumina sequencing and mapping. Total reads: The total amount of clean reads. Total mapped: The total amount of reads mapped to the reference genome/sequence. Multiple mapped: The amount of reads mapped to the reference genome/sequence at more than one site. Uniquely mapped: The amount of reads mapped to the reference genome/sequence at only one site.
(DOCX)

S7 Table Classification of raw reads. (DOCX)

S8 Table Differentially expressed lncRNAs in duodenum tissues of *E*. *coli* F18-resistant and sensitive individuals from Meishan piglets. Fold change means *E*. *coli* F18-resistant group/*E*. *coli* F18-sensitive group. MR: Meishan F18-resistant piglets; MS: Meishan F18-sensitive piglets.
(DOCX)

S9 Table Differentially expressed lncRNAs in duodenum tissues of *E*. *coli* F18-resistant and sensitive individuals from Sutai piglets. Fold change means *E*. *coli* F18-resistant group/*E*. *coli* F18-sensitive group. SR: Sutai F18-resistant piglets; SS: Sutai F18-sensitive piglets.
(DOCX)

S10 Table Predicted targets of three common lncRNAs in Meishan and Sutai piglets. (DOCX)

S11 Table Identification of FUT3-AS1-associated proteins using RNA pull down and mass spectrometry. K represents *E*. *coli* F18-resistant piglets; M represents *E*. *coli* F18-sensitive piglets.
(DOCX)

S12 Table Statistics of differentially expressed miRNAs between Sutai *E*. *coli* F18-resistant and -sensitive piglets. (DOCX)

S13 Table ceRNA construction of FUT3-AS1 (TCONS_00183659)-miRNAs-*FUT3*. (DOCX)

S14 Table Key differentially abundant proteins between the pig siFUT3-AS1 group and the control group IPEC-J2 cells based on iTRAQ proteome analysis. (DOCX)
