# Supplementary figures and images for: Insight into mechanisms of pig lncRNA FUT3-AS1 regulating E. coli F18-bacterial diarrhea
Source: PLoS Pathog. 2022 Jun 13;18(6):e1010584. doi: 10.1371/journal.ppat.1010584 (PMC9191744; doi:10.1371/journal.ppat.1010584)

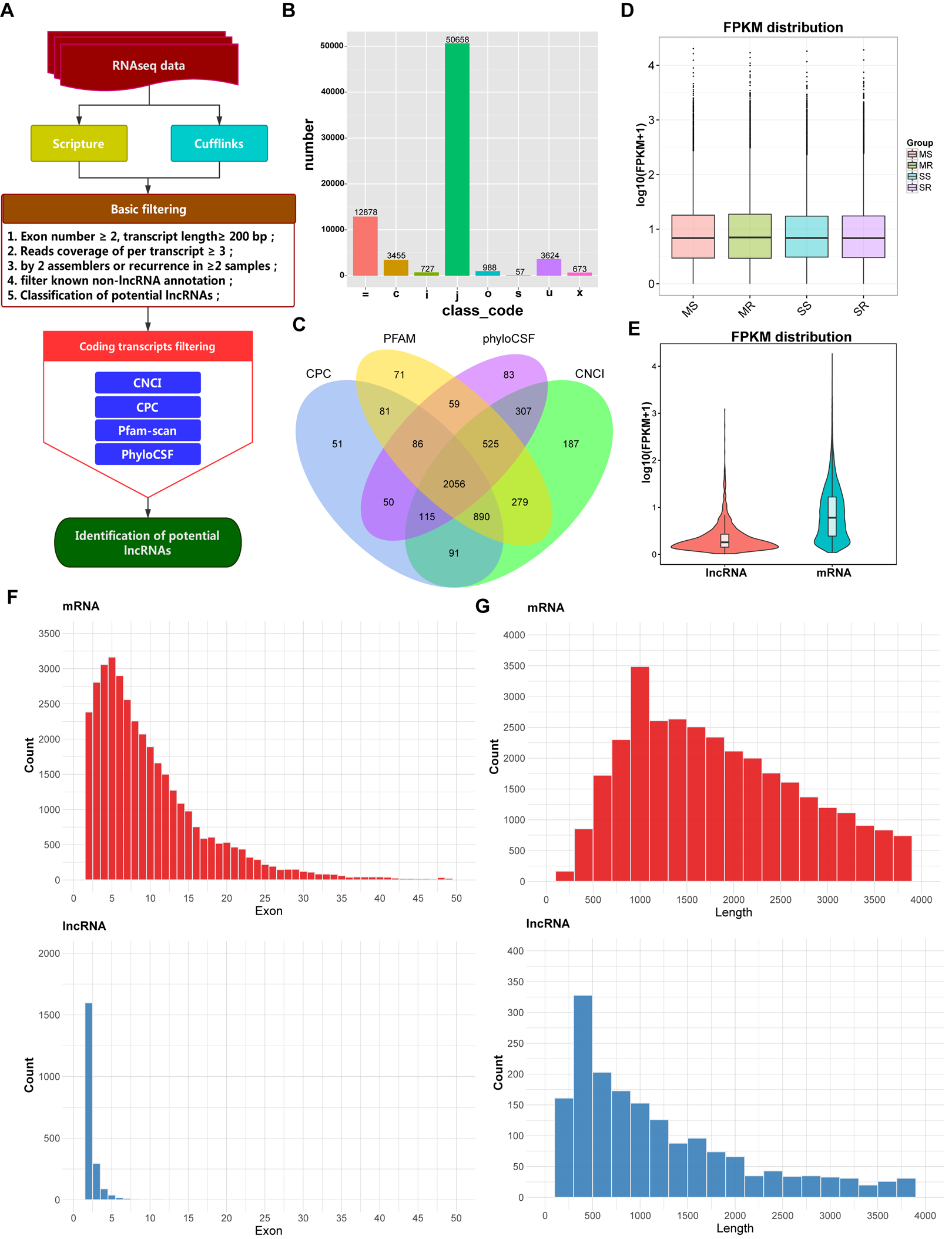

Supplement: S1 Fig — (A) The screening workflow for potential lncRNAs. (B) lncRNA classifications. ‘x’, ‘u’, ‘i’, and ‘o’ represent antisense lncRNA, intergenic lncRNA (lincRNA), intronic lncRNA, and sense lncRNA, respectively. ‘ = ‘ for complete match of intron chain, ‘c’ for contained, ‘j’ for potentially novel isoform, ‘s’ for an intron of the transfrag overlaps a reference intron on the opposite strand. (C) Coding potential analysis of lncRNAs based on four computational approaches (CNCI, CPC, PFAM, and PhyloCSF). (D) The FPKM distribution is shown in a box plot, showing no significant differences among the different groups. (E) Expression levels of lncRNAs and mRNAs are shown as a violin plot, showing certain differences between lncRNAs and mRNAs. (F) Structural comparison between lncRNAs and mRNAs in terms of exon number. (G) LncRNAs and mRNAs transcript length comparison. (TIF) [file ppat.1010584.s002.tif]

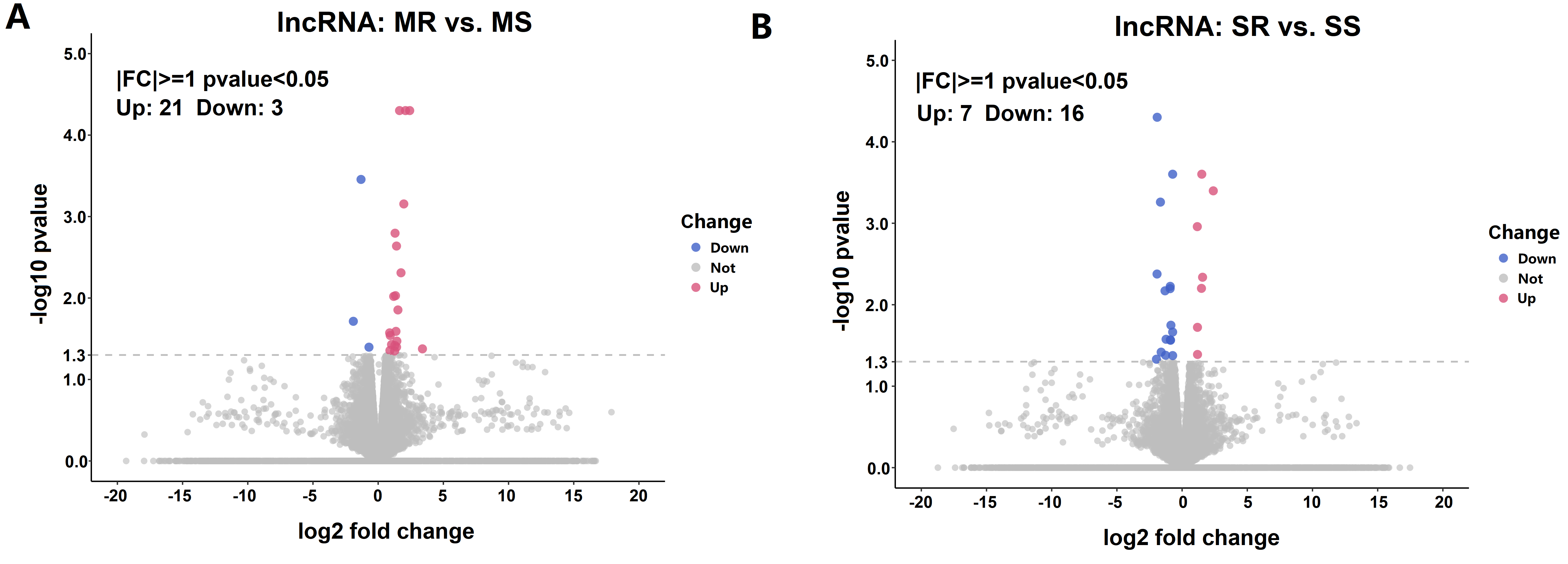

Supplement: S2 Fig — (A) Volcano plot analysis of differential expression lncRNAs between Meishan F18-resistant and -sensitive piglets. (B) Volcano plot analysis of differential expression lncRNAs between Sutai F18-resistant and -sensitive piglets. (TIF) [file ppat.1010584.s003.tif]

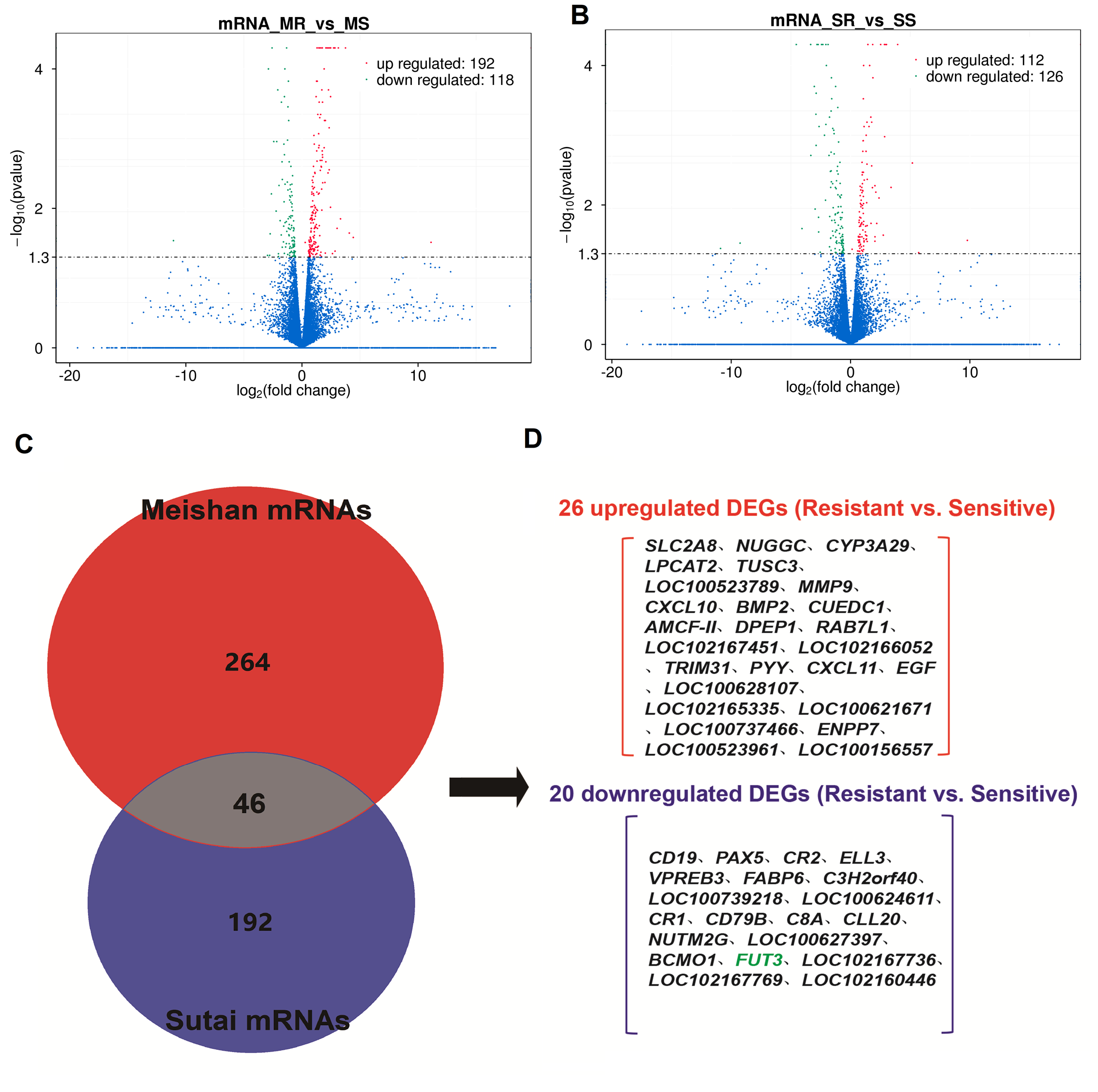

Supplement: S3 Fig — (A) Volcano plot analysis of differential expression mRNAs between Meishan F18-resistant and -sensitive piglets. (B) Volcano plot analysis of differential expression mRNAs between Sutai F18-resistant and -sensitive piglets. (C) Venn diagram screening of host mRNAs related to E. coli F18 infection. (D) Common differentially expressed genes (DEGs) between E. coli F18-resistant and sensitive individuals from Meishan and Sutai piglets. (TIF) [file ppat.1010584.s004.tif]

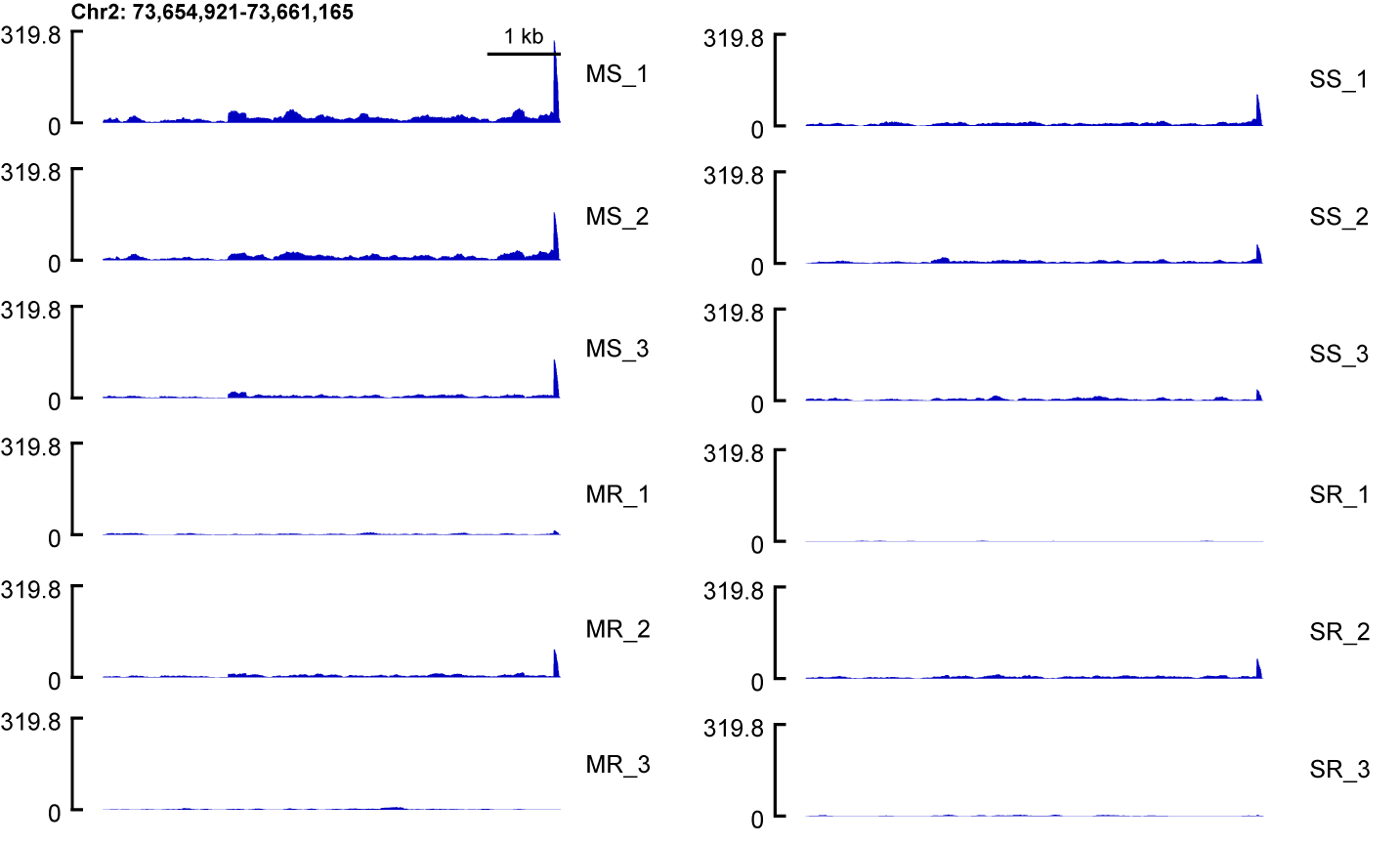

Supplement: S4 Fig — MS represents Meishan F18-sensitive piglets (n = 3); MR represents Meishan F18-resistant piglets (n = 3); SS represents Sutai F18-sensitive piglets (n = 3); SR represents Sutai F18-resistant piglets (n = 3). (TIF) [file ppat.1010584.s005.tif]

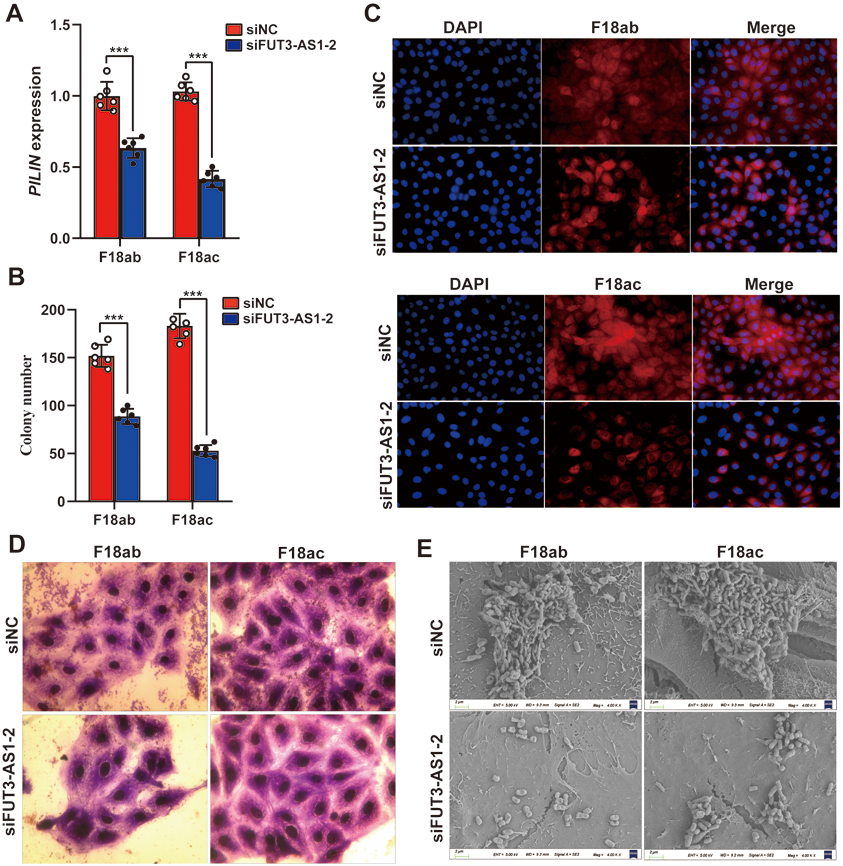

Supplement: S5 Fig — (A) Expression detection of E. coi F18 fimbriae gene (PILIN) via relative quantification in FUT3-AS1-silenced IPEC-2 cells. (B) Colony number of E. coi F18 fimbria adhering to IPEC-J2 cells were evaluated, n = 6, mean ± SEM, ***P<0.001. (C) Immunofluorescence assay, blue fluorescence indicates nuclear staining via DAPI; red fluorescence indicates staining with the anti-E. coli antibody. Cells were observed under a fluorescence microscope (100×). (D) Gram staining assay, an optical microscope (400×) was used to observe the cells. (E) Scanning electron microscopy (SEM) assay, cells were observed under a scanning electron microscope (4000×). (TIF) [file ppat.1010584.s006.tif]

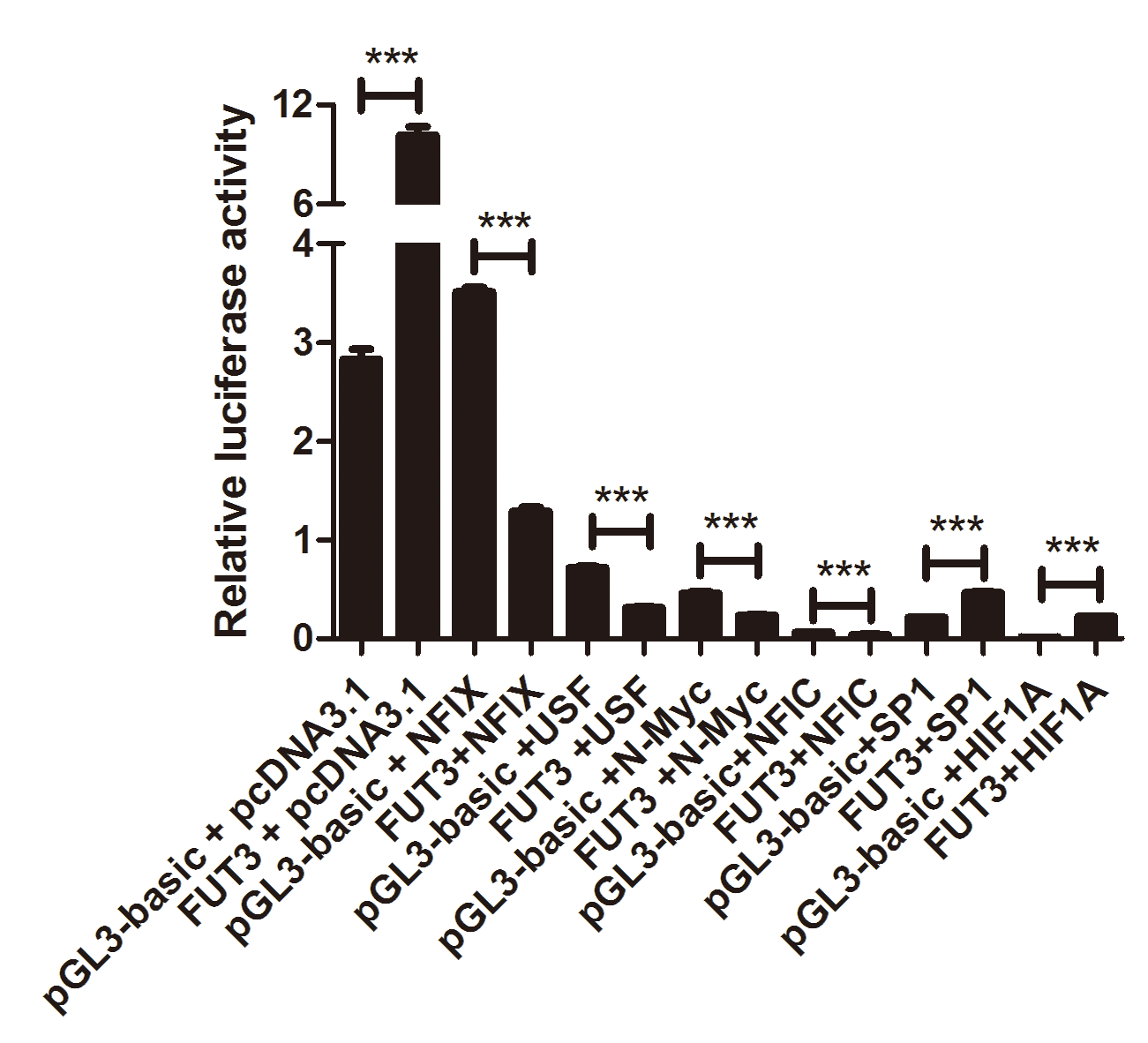

Supplement: S6 Fig — Transcriptional activity determination of NFIC, NFIX, SP1, USF, HIF1A, and N-Myc in the FUT3 core promoter using a dual-luciferase assay. The obtained results are indicated as the mean ± SEM, **P<0.01. (TIF) [file ppat.1010584.s007.tif]

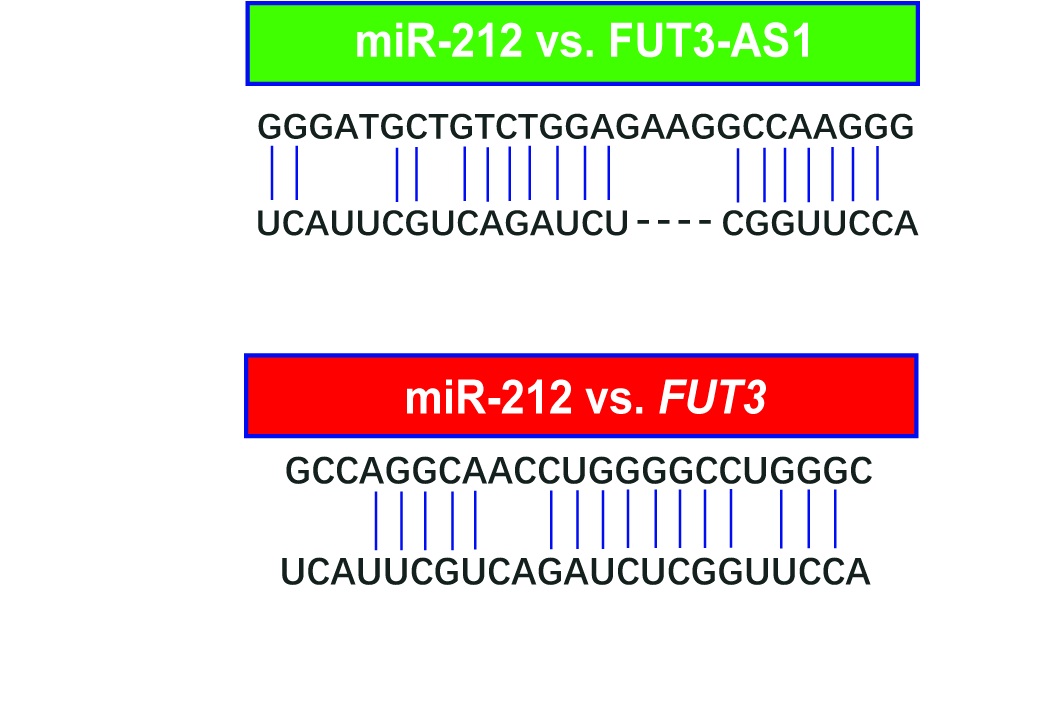

Supplement: S7 Fig — (TIF) [file ppat.1010584.s008.tif]

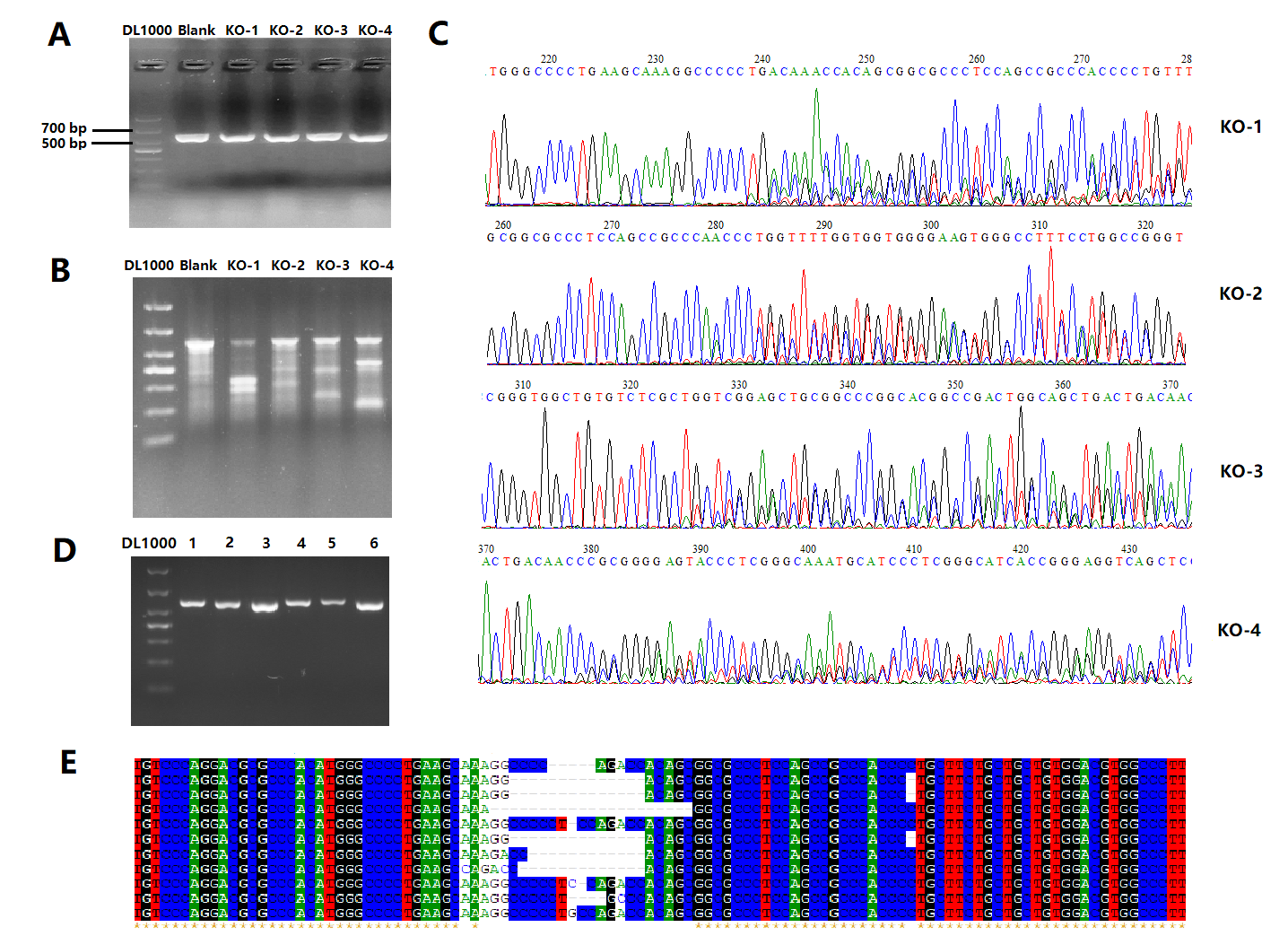

Supplement: S8 Fig — (A) PCR results of knockout vector transfected cells, where Blank represents blank cells, and KO-1~4 are cells transfected with different knockout vectors. (B) Digestion results of PCR products of knockout vector transfected cells. (C) PCR sequencing results of knockout vector-transfected cells. (D) knockout single cell PCR results, where 1 represents blank cells, 2~6 represents the knockout single cells. (E) PCR sequencing results of the transfected cells with the knockout vector; the last line represents blank untreated cells. (TIF) [file ppat.1010584.s009.tif]

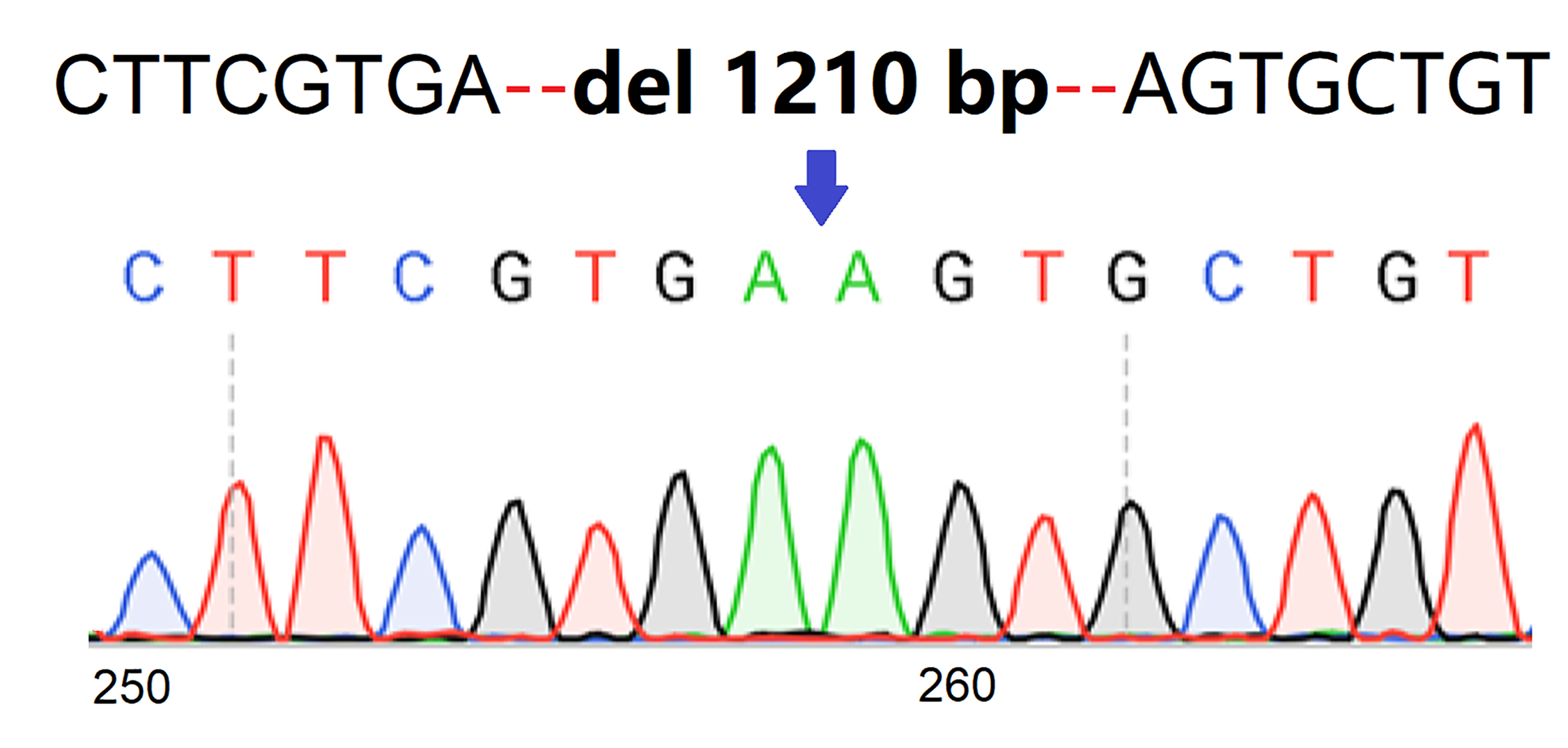

Supplement: S9 Fig — Fut3 deletion fragment (1210 bp) was identified by sequencing. (TIF) [file ppat.1010584.s010.tif]

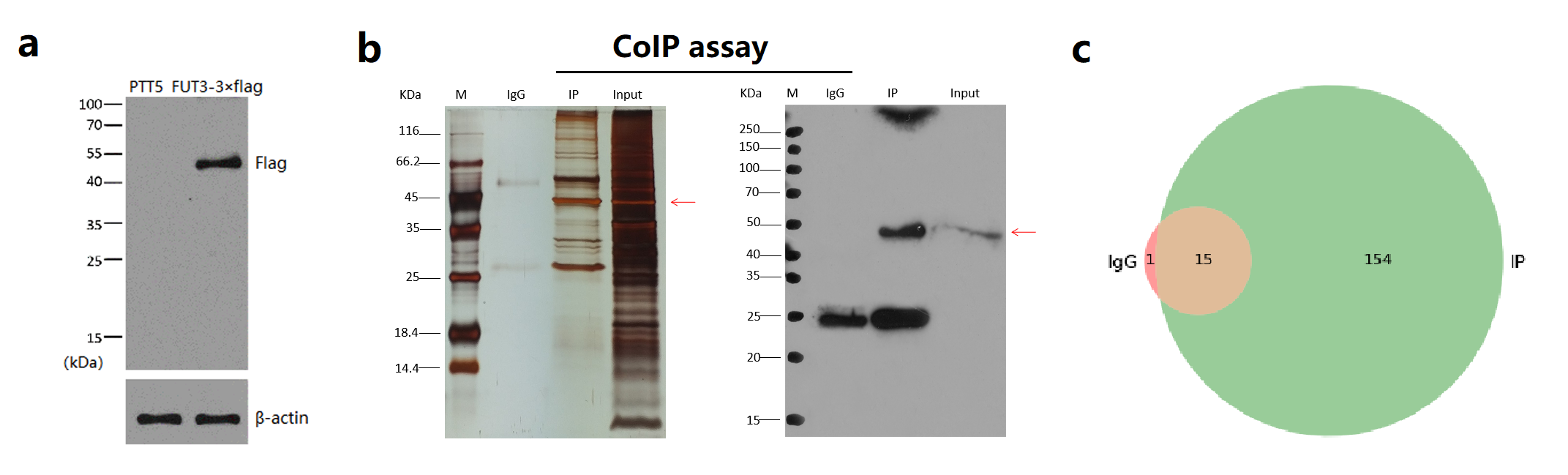

Supplement: S10 Fig — (a) Validation of the expression of the FUT3-flag protein by western blotting. (b) CoIP assay results. (c) Numbers of proteins identified using mass spectrometry. (TIF) [file ppat.1010584.s011.tif]

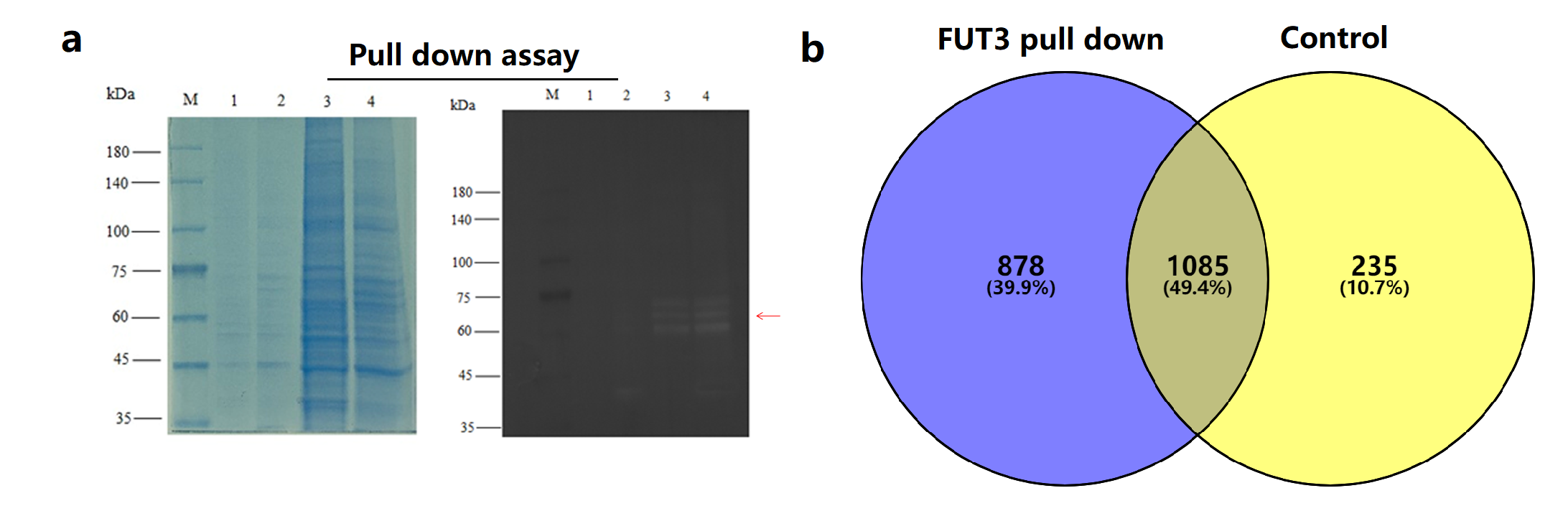

Supplement: S11 Fig — (a) His-pull down assay results. (b) Numbers of proteins identified using mass spectrometry. (TIF) [file ppat.1010584.s012.tif]
