# Supplementary material for: Insight into mechanisms of pig lncRNA FUT3-AS1 regulating E. coli F18-bacterial diarrhea
Source: PLoS Pathog. 2022 Jun 13;18(6):e1010584. doi: 10.1371/journal.ppat.1010584 (PMC9191744; doi:10.1371/journal.ppat.1010584)
Supplement: S1 Table — (DOCX) [file ppat.1010584.s013.docx]

**S1 Table. Primer sequences of pig FUT3-AS1 siRNAs**

| Name | Sequence (5'→3') |
| --- | --- |
| siFUT3-AS1-1F | CCCUGUGAAGUGACAGGAUTT |
| siFUT3-AS1-1R | AUCCUGUCACUUCACAGGGTT |
| siFUT3-AS1-2F | GCUAGAACUCCCAGGGAAUTT |
| siFUT3-AS1-2R | AUUCCCUGGGAGUUCUAGCTT |
| siNC-F | UUCUCCGAACGUGUCACGUTT |
| siNC-R | ACGUGACACGUUCGGAGAATT |
