# Supplementary material for: Insight into mechanisms of pig lncRNA FUT3-AS1 regulating E. coli F18-bacterial diarrhea
Source: PLoS Pathog. 2022 Jun 13;18(6):e1010584. doi: 10.1371/journal.ppat.1010584 (PMC9191744; doi:10.1371/journal.ppat.1010584)
Supplement: S2 Table — (DOCX) [file ppat.1010584.s014.docx]

**S2 Table. Primer sequences of miR-212 mimics and inhibitor**

| Name | Sense (5'→3') | Antisense (5'→3') |
| --- | --- | --- |
| miR-212 mimics | ACCUUGGCUCUAGACUGCUUACU | AGUAAGCAGUCUAGAGCCAAGGU |
| mimics NC | UCACAACCUCCUAGAAAGAGUAGA | UCUACUCUUUCUAGGAGGUUGUGA |
| miR-212 inhibitor | AGUAAGCAGUCUAGAGCCAAGGU |  |
| inhibitor NC | UCUACUCUUUCUAGGAGGUUGUGA |  |
