# Supplementary material for: Insight into mechanisms of pig lncRNA FUT3-AS1 regulating E. coli F18-bacterial diarrhea
Source: PLoS Pathog. 2022 Jun 13;18(6):e1010584. doi: 10.1371/journal.ppat.1010584 (PMC9191744; doi:10.1371/journal.ppat.1010584)
Supplement: S3 Table — (DOCX) [file ppat.1010584.s015.docx]

**S3 Table. Single-strand DNA sequences for pig *FUT3* knockout**

| Name | Sequence (5'→3') |
| --- | --- |
| KO-1F | caccGGCGCCGCTGTGGTCTGGCAG |
| KO-1R | aaacCTGCCAGACCACAGCGGCGCC |
| KO-2F | caccGGCAGCAGAAGCAGGGGTGGG |
| KO-2R | aaacCCCACCCCTGCTTCTGCTGCC |
| KO-3F | caccGGGTGGCTGTGTCTCGCTGCT |
| KO-3R | aaac AGCAGCGAGACACAGCCACCC |
| KO-4F | caccGCCAGCTGACTGACAACCGCG |
| KO-4R | aaacCGCGGTTGTCAGTCAGCTGGC |
