# Supplementary material for: Insight into mechanisms of pig lncRNA FUT3-AS1 regulating E. coli F18-bacterial diarrhea
Source: PLoS Pathog. 2022 Jun 13;18(6):e1010584. doi: 10.1371/journal.ppat.1010584 (PMC9191744; doi:10.1371/journal.ppat.1010584)
Supplement: S4 Table — (DOCX) [file ppat.1010584.s016.docx]

**S4 Table. Real-time PCR primers and sequences**

| Gene name | GenBank  accession number | Primer sequence | Fragment Size (bp) |
| --- | --- | --- | --- |
| *FUT2* | U70881.2 | F: 5′-AATCCCTGACCTCACTCCGTG-3′ | 123 |
|  |  | R: 5′-CGGAACTACAACTGCTGGCC-3′ |  |
| *ST3GAL3* | NM_213759.1 | F: 5′-TCCAAACCAGCACCCATGTT-3′ | 126 |
|  |  | R: 5′-TTTGGTGACGGACAGGATGG-3′ |  |
| *B3GALNT1* | NM_214351.1 | F: 5′-GCTAAGGAACACCACATGCCA-3′ | 137 |
|  |  | R: 5′-GCGACTCAGTGACCTTCCCAT-3’ |  |
| *FUT3* | AF130972.1 | F: 5′-CCCGAAGCCTTCATCCACAT-3′ | 150 |
|  |  | R: 5′-CATCAAGGCCCAGCTGAAGA-3′ |  |
| *FUT3-AS1* | XLOC_017083 | F: 5′- CTGAGACGGAACAAGGAGGT-3′ | 174 |
|  |  | R: 5′-GGCGTAAAGTTGACCCATCC-3′ |  |
| *TNF-α* | JF831365.1 | F: 5′-CCTACTGCACTTCGAGGTTATC-3′ | 158 |
|  |  | R: 5′-GCATACCCACTCTGCCATT-3′ |  |
| *IL-6* | JQ839263.1 | F: 5′-CTCTGTCTTAGGGCGTCC-3′ | 164 |
|  |  | R: 5′-CAAGGAGGTACTGGCAGAAA-3′ |  |
| *IL-12* | NM_213993.1 | F: 5′-CAGGCCCAGGAATGTTCAAA-3′ | 166 |
|  |  | R:5′- CGTGGCTAGTTCAAGTGGTAAG-3′ |  |
| *IL-1β* | XM_021085847.1 | F: 5′-CACTTCTGGTGTGGGCTTAAC-3′ | 283 |
|  |  | R: 5′-CAGGTCATTATTGTTGTCACCGT-3′ |  |
| *MyD88* | NM_001099923.1 | F:5′-GTGCCGTCGGATGGTAGT-3′ | 173 |
|  |  | R:5′-CAGTGATGAACCGCAGGAT-3′ |  |
| *LRRFIP2* | XM_021071626.2 | F: 5′-GCTGAAAACAGTGCAGTTCGT-3′ | 225 |
|  |  | R: 5′-TCTGTTGCCGTTCCAGTTCTC-3′ |  |
| *TRAF6* | NM_001105286.1 | F: 5′-GCTGCCATGAAAAGATGCAGAG-3′ | 244 |
|  |  | R: 5′-TGGTCCTGTCTTACAAGGCG-3′ |  |
| *GAPDH* | XM_021091114.1 | F: 5′-ACTCACTCTTCTACCTTTGATGCTG-3′ | 100 |
|  |  | R: 5′-TGTTGCTGTAGCCAAATTCA-3′ |  |
| *ACTB* | NC_010445.3 | F: 5′-GTCGTACTCCTGCTTGCTGAT-3′  R: 5′-CCTTCTCCTTCCAGATCATCGC-3′ | 119 |
