# Supplementary material for: Insight into mechanisms of pig lncRNA FUT3-AS1 regulating E. coli F18-bacterial diarrhea
Source: PLoS Pathog. 2022 Jun 13;18(6):e1010584. doi: 10.1371/journal.ppat.1010584 (PMC9191744; doi:10.1371/journal.ppat.1010584)
Supplement: S5 Table — (DOCX) [file ppat.1010584.s017.docx]

**S5 Table.** Sequencing data quality summary

| Sample | Raw reads | Clean reads | clean bases | Error rate (%) | Q20 (%) | Q30 (%) | GC content (%) |
| --- | --- | --- | --- | --- | --- | --- | --- |
| MR_1 | 77323749 | 70953091 | 7.1 Gb | 0.04 | 95.78 | 88.23 | 49.56 |
| MR_2 | 63966176 | 54082572 | 5.41 Gb | 0.05 | 95.47 | 87.61 | 51.11 |
| MR_3 | 72540039 | 67098831 | 6.71 Gb | 0.03 | 98.23 | 94.75 | 46.92 |
| MS_1 | 70465906 | 63307920 | 6.33 Gb | 0.05 | 95.52 | 87.78 | 50.58 |
| MS_2 | 67381338 | 57403813 | 5.74 Gb | 0.05 | 95.81 | 88.18 | 51.1 |
| MS_3 | 68881505 | 60727146 | 6.07 Gb | 0.04 | 95.64 | 88.37 | 48.85 |
| SR_1 | 73564342 | 66231494 | 6.62 Gb | 0.03 | 96.62 | 90.61 | 50.28 |
| SR_2 | 77140894 | 70258719 | 7.03 Gb | 0.05 | 95.57 | 87.77 | 50.46 |
| SR_3 | 73694573 | 66092615 | 6.61 Gb | 0.03 | 97.67 | 93.62 | 50.39 |
| SS_1 | 77712262 | 73248077 | 7.32 Gb | 0.05 | 94.83 | 85.26 | 51.57 |
| SS_2 | 75635464 | 68599576 | 6.86 Gb | 0.05 | 95.67 | 88 | 50.42 |
| SS_3 | 71775610 | 63321583 | 6.33 Gb | 0.04 | 96.01 | 88.95 | 47.67 |
