# Supplementary material for: Insight into mechanisms of pig lncRNA FUT3-AS1 regulating E. coli F18-bacterial diarrhea
Source: PLoS Pathog. 2022 Jun 13;18(6):e1010584. doi: 10.1371/journal.ppat.1010584 (PMC9191744; doi:10.1371/journal.ppat.1010584)
Supplement: S6 Table — Total reads: The total amount of clean reads. Total mapped: The total amount of reads mapped to the reference genome/sequence. Multiple mapped: The amount of reads mapped to the reference genome/sequence at more than one site. Uniquely mapped: The amount of reads mapped to the reference genome/sequence at only one site. (DOCX) [file ppat.1010584.s018.docx]

**S6 Table. Summary of Illumina sequencing and mapping** (a)

| Sample name | MR_1 | MR_2 | MR_3 | MS_1 | MS_2 | MS_3 |
| --- | --- | --- | --- | --- | --- | --- |
| Total reads | 1.42E+08 | 1.08E+08 | 1.34E+08 | 1.27E+08 | 1.15E+08 | 1.21E+08 |
| Total mapped | 118429897 (83.46%) | 87577707 (80.97%) | 108561453 (80.9%) | 102959244 (81.32%) | 92530851 (80.6%) | 91176887 (75.07%) |
| Multiple mapped | 16014918 (11.29%) | 12348679 (11.42%) | 15144257 (11.29%) | 16312123 (12.88%) | 14137532 (12.31%) | 11970240 (9.86%) |
| Uniquely mapped | 102414979 (72.17%) | 75229028 (69.55%) | 93417196 (69.61%) | 86647121 (68.43%) | 78393319 (68.28%) | 79206647 (65.22%) |

**S6 Table. Summary of Illumina sequencing and mapping** (b)

| Sample name | SR_1 | SR_2 | SR_3 | SS_1 | SS_2 | SS_3 |
| --- | --- | --- | --- | --- | --- | --- |
| Total reads | 132462988 | 140517438 | 132185230 | 146496154 | 137199152 | 126643166 |
| Total mapped | 109548356 (82.7%) | 115637106 (82.29%) | 107632615 (81.43%) | 119502981 (81.57%) | 113359916 (82.62%) | 102952535 (81.29%) |
| Multiple mapped | 16283456 (12.29%) | 18551739 (13.2%) | 15673712 (11.86%) | 19132602 (13.06%) | 18512786 (13.49%) | 11973368 (9.45%) |
| Uniquely mapped | 93264900 (70.41%) | 97085367 (69.09%) | 91958903 (69.57%) | 100370379 (68.51%) | 94847130 (69.13%) | 90979167 (71.84%) |

Total reads: The total amount of clean reads. Total mapped: The total amount of reads mapped to the reference genome/sequence. Multiple mapped: The amount of reads mapped to the reference genome/sequence at more than one site. Uniquely mapped: The amount of reads mapped to the reference genome/sequence at only one site.
