# Supplementary material for: Insight into mechanisms of pig lncRNA FUT3-AS1 regulating E. coli F18-bacterial diarrhea
Source: PLoS Pathog. 2022 Jun 13;18(6):e1010584. doi: 10.1371/journal.ppat.1010584 (PMC9191744; doi:10.1371/journal.ppat.1010584)
Supplement: S7 Table — (DOCX) [file ppat.1010584.s019.docx]

**S7 Table. Classification of raw reads** (a)

| Samples | MS_1 | MS_2 | MS_3 | MR_1 | MR_2 | MR_3 |
| --- | --- | --- | --- | --- | --- | --- |
| mRNA | 58266534 (69.93%) | 52872771 (69.59%) | 43304782 (55.86%) | 68634434 (69.29%) | 51242964 (70.45%) | 56071697 (61.81%) |
| misc_RNA | 2409369 (2.89%) | 2143891 (2.82%) | 2440127 (3.15%) | 2561882 (2.59%) | 2222788 (3.06%) | 2974516 (3.28%) |
| ncRNA | 301  (0.00%) | 302  (0.00%) | 281  (0.00%) | 348  (0.00%) | 234  (0.00%) | 380  (0.00%) |
| precursor_RNA | 1320  (0.00%) | 880 (0.00%) | 516 (0.00%) | 1140(0.00%) | 890 (0.00%) | 873 (0.00%) |
| pseudogene | 151331 (0.18%) | 164152 (0.22%) | 201966 (0.26%) | 162718 (0.16%) | 157054 (0.22%) | 211390 (0.23%) |
| rRNA | 349365 (0.42%) | 189690 (0.25%) | 498879 (0.64%) | 2439962 (2.46%) | 145328 (0.20%) | 312212 (0.34%) |
| tRNA | 7740  (0.01%) | 8178 (0.01%) | 13121 (0.02%) | 8039  (0.01%) | 5213 (0.01%) | 15775 (0.02%) |
| Others | 22135202 (26.57%) | 20596910 (27.11%) | 31066521 (40.07%) | 25252367 (25.49%) | 18960367 (26.07%) | 31131581 (34.32%) |

**S7 Table. Classification of raw reads** (b)

| Samples | SS_1 | SS_2 | SS_3 | SR_1 | SR_2 | SR_3 |
| --- | --- | --- | --- | --- | --- | --- |
| mRNA | 61561316 (63.00%) | 61737961 (66.91%) | 54746527 (61.64%) | 60335228 (66.54%) | 62529055 (66.44%) | 60529934 (67.61%) |
| misc_RNA | 2889617 (2.96%) | 2774389 (3.01%) | 2771658 (3.12%) | 2843749 (3.14%) | 2820694 (3.00%) | 2749765 (3.07%) |
| ncRNA | 467  (0.00%) | 418  (0.00%) | 504  (0.00%) | 825  (0.00%) | 486  (0.00%) | 373  (0.00%) |
| precursor_RNA | 1250 (0.00%) | 1479 (0.00%) | 668 (0.00%) | 1624 (0.00%) | 1268 (0.00%) | 1144 (0.00%) |
| pseudogene | 224670 (0.23%) | 221146 (0.24%) | 203599 (0.23%) | 202124 (0.22%) | 215183 (0.23%) | 182008 (0.20%) |
| rRNA | 274484 (0.28%) | 258050 (0.28%) | 1341517 (1.51%) | 324578 (0.36%) | 237461 (0.25%) | 338546 (0.38%) |
| tRNA | 5742 (0.01%) | 7275 (0.01%) | 9651 (0.01%) | 5754 (0.01%) | 7122 (0.01%) | 14852 (0.02%) |
| Others | 32757350 (33.52%) | 27272154 (29.56%) | 29748872 (33.49%) | 26966373 (29.74%) | 28298895 (30.07%) | 25715988 (28.72%) |
