# Supplementary material for: Insight into mechanisms of pig lncRNA FUT3-AS1 regulating E. coli F18-bacterial diarrhea
Source: PLoS Pathog. 2022 Jun 13;18(6):e1010584. doi: 10.1371/journal.ppat.1010584 (PMC9191744; doi:10.1371/journal.ppat.1010584)
Supplement: S8 Table — Fold change means E. coli F18-resistant group/E. coli F18-sensitive group. MR: Meishan F18-resistant piglets; MS: Meishan F18-sensitive piglets. (DOCX) [file ppat.1010584.s020.docx]

**S8 Table. Differentially expressed lncRNAs in duodenum tissues of *E. coli* F18-resistant and sensitive individuals from Meishan piglets.**

| transcript_id | gene_id | length | MR_FPKM | MS_FPKM | log2 (foldchange) | p-value |
| --- | --- | --- | --- | --- | --- | --- |
| TCONS_00127256 | XLOC_011338 | 1145 | 0.273101 | 1.02318 | −1.90555 | 0.0193 |
| TCONS_00183659 | XLOC_017083 | 5833 | 1.1377 | 2.83128 | −1.31533 | 0.00035 |
| TCONS_00088547 | XLOC_009550 | 3186 | 1.77847 | 2.89954 | −0.705184 | 0.0401 |
| TCONS_00232414 | XLOC_020029 | 2827 | 1.70991 | 0.928925 | 0.88028 | 0.02675 |
| TCONS_00268366 | XLOC_024324 | 4798 | 0.686555 | 0.367818 | 0.90038 | 0.044 |
| TCONS_00259409 | XLOC_021718 | 4107 | 8.46168 | 4.45603 | 0.92519 | 0.0288 |
| TCONS_00326977 | XLOC_026682 | 3241 | 1.00258 | 0.492706 | 1.02492 | 0.03725 |
| TCONS_00254248 | XLOC_021270 | 1279 | 4.65908 | 2.04231 | 1.18985 | 0.00955 |
| TCONS_00353682 | XLOC_029466 | 1250 | 1.14465 | 0.476392 | 1.26469 | 0.0447 |
| TCONS_00315896 | XLOC_027140 | 2716 | 0.518285 | 0.21336 | 1.28046 | 0.0382 |
| TCONS_00255937 | XLOC_021877 | 4248 | 10.4775 | 4.27084 | 1.2947 | 0.0016 |
| TCONS_00155440 | XLOC_013828 | 4530 | 0.801459 | 0.319116 | 1.32855 | 0.00935 |
| TCONS_00268463 | XLOC_024436 | 833 | 3.41216 | 1.32558 | 1.36407 | 0.02565 |
| TCONS_00315894 | XLOC_027139 | 1839 | 2.57672 | 0.972084 | 1.40638 | 0.0023 |
| TCONS_00073969 | XLOC_007903 | 6804 | 2.28236 | 0.858854 | 1.41004 | 0.04 |
| TCONS_00352684 | XLOC_029470 | 1884 | 0.648869 | 0.240266 | 1.4333 | 0.0338 |
| TCONS_00352975 | XLOC_029383 | 6095 | 76.4883 | 26.7512 | 1.51563 | 0.014 |
| TCONS_00087233 | XLOC_010344 | 1129 | 79.4163 | 25.5098 | 1.63838 | 5.00E-05 |
| TCONS_00163002 | XLOC_014574 | 1913 | 7.77208 | 2.30864 | 1.75126 | 0.0049 |
| TCONS_00087234 | XLOC_010344 | 1335 | 14.0757 | 3.60197 | 1.96635 | 0.0007 |
| TCONS_00019308 | XLOC_004904 | 14682 | 0.652833 | 0.15266 | 2.09639 | 5.00E-05 |
| TCONS_00053650 | XLOC_006029 | 3430 | 3.32844 | 0.622463 | 2.41879 | 5.00E-05 |
| TCONS_00254202 | XLOC_021176 | 1058 | 0.959691 | 0.0911758 | 3.39585 | 0.04205 |
| TCONS_00359118 | XLOC_029609 | 980 | 0.592624 | 0 | inf | 5.00E-05 |

Fold change means *E. coli* F18-resistant group/*E. coli* F18-sensitive group. MR: Meishan F18-resistant piglets; MS: Meishan F18-sensitive piglets.
