# Supplementary material for: Insight into mechanisms of pig lncRNA FUT3-AS1 regulating E. coli F18-bacterial diarrhea
Source: PLoS Pathog. 2022 Jun 13;18(6):e1010584. doi: 10.1371/journal.ppat.1010584 (PMC9191744; doi:10.1371/journal.ppat.1010584)
Supplement: S9 Table — Fold change means E. coli F18-resistant group/E. coli F18-sensitive group. SR: Sutai F18-resistant piglets; SS: Sutai F18-sensitive piglets. (DOCX) [file ppat.1010584.s021.docx]

**S9 Table. Differentially expressed lncRNAs in duodenum tissues of *E. coli* F18-resistant and sensitive individuals from Sutai piglets.**

| transcript_id | gene_id | length | SR_FPKM | SS_FPKM | log2 (foldchange) | p-value |
| --- | --- | --- | --- | --- | --- | --- |
| TCONS_00354297 | XLOC_029233 | 976 | 0.180345 | 0.705262 | −1.9674 | 0.04665 |
| TCONS_00250742 | XLOC_021876 | 3282 | 0.170605 | 0.646436 | −1.92185 | 0.0042 |
| TCONS_00287947 | XLOC_023383 | 6858 | 0.260486 | 0.979366 | −1.91064 | 5.00E-05 |
| TCONS_00183659 | XLOC_017083 | 5833 | 0.566717 | 1.79285 | −1.66155 | 0.00055 |
| TCONS_00025901 | XLOC_004924 | 1280 | 0.279401 | 0.850669 | −1.60626 | 0.0382 |
| TCONS_00263081 | XLOC_021562 | 1616 | 0.884582 | 2.19363 | −1.31025 | 0.00675 |
| TCONS_00228897 | XLOC_018238 | 1703 | 0.375159 | 0.903137 | −1.26744 | 0.04175 |
| TCONS_00136647 | XLOC_011513 | 2891 | 0.308832 | 0.724474 | −1.23011 | 0.02655 |
| TCONS_00267254 | XLOC_023218 | 18283 | 0.878575 | 1.66188 | −0.919583 | 0.00635 |
| TCONS_00057623 | XLOC_006721 | 3227 | 2.11504 | 3.97604 | −0.910644 | 0.00595 |
| TCONS_00221163 | XLOC_018046 | 934 | 3.14245 | 5.88024 | -0.903985 | 0.027 |
| TCONS_00330080 | XLOC_027377 | 4300 | 1.00182 | 1.85136 | −0.885971 | 0.0273 |
| TCONS_00053650 | XLOC_006029 | 3430 | 1.52875 | 2.77074 | −0.857919 | 0.01785 |
| TCONS_00304609 | XLOC_024647 | 1583 | 18.9119 | 31.2628 | −0.725149 | 0.02165 |
| TCONS_00346329 | XLOC_028358 | 452 | 0 | 5.81028 | #NAME? | 0.0421 |
| TCONS_00152864 | XLOC_012706 | 429 | 0 | 0.843687 | #NAME? | 0.00025 |
| TCONS_00208724 | XLOC_018306 | 4673 | 2.20067 | 0.980455 | 1.16642 | 0.0011 |
| TCONS_00241482 | XLOC_020160 | 5680 | 0.545579 | 0.241025 | 1.1786 | 0.01895 |
| TCONS_00238677 | XLOC_019774 | 2108 | 0.746526 | 0.329081 | 1.18175 | 0.0408 |
| TCONS_00352975 | XLOC_029383 | 6095 | 46.9432 | 16.768 | 1.4852 | 0.0063 |
| TCONS_00066754 | XLOC_006973 | 2104 | 7.09476 | 2.49182 | 1.50955 | 0.00025 |
| TCONS_00175609 | XLOC_014833 | 1052 | 2.84354 | 0.957594 | 1.5702 | 0.0046 |
| TCONS_00338218 | XLOC_027444 | 1573 | 2.10515 | 0.400988 | 2.39229 | 0.0004 |

Fold change means *E. coli* F18-resistant group/*E. coli* F18-sensitive group. SR: Sutai F18-resistant piglets; SS: Sutai F18-sensitive piglets.
