# Supplementary material for: Insight into mechanisms of pig lncRNA FUT3-AS1 regulating E. coli F18-bacterial diarrhea
Source: PLoS Pathog. 2022 Jun 13;18(6):e1010584. doi: 10.1371/journal.ppat.1010584 (PMC9191744; doi:10.1371/journal.ppat.1010584)
Supplement: S10 Table — (DOCX) [file ppat.1010584.s022.docx]

**S10 Table. Predicted targets of three common lncRNAs in Meishan and Sutai piglets.**

| lncRNA_id | lncRNA_id | cisGene_id | Target_gene_name |
| --- | --- | --- | --- |
| TCONS_00352975 | XLOC_029383 | 100526199 | *CHIC1* |
| TCONS_00183659 | XLOC_017083 | 100516593 | *RFX2* |
| TCONS_00183659 | XLOC_017083 | 100513645 | *NRTN* |
| TCONS_00183659 | XLOC_017083 | 100513844 | *FUT3* |
| TCONS_00183659 | XLOC_017083 | 100514033 | *NDUFA11* |
| TCONS_00183659 | XLOC_017083 | 100514213 | *VMAC* |
| TCONS_00183659 | XLOC_017083 | 100514564 | *CAPS* |
| TCONS_00183659 | XLOC_017083 | 100514391 | *RANBP3* |
| TCONS_00183659 | XLOC_017083 | 100513458 | *DUS3L* |
| TCONS_00183659 | XLOC_017083 | 100513267 | *PRR22* |
| TCONS_00183659 | XLOC_017083 | 100516061 | *CATSPERD* |
| TCONS_00053650 | XLOC_006029 | 100623097 | *SLC30A10* |
| TCONS_00053650 | XLOC_006029 | 449526 | *EPRS* |
