# Supplementary material for: Insight into mechanisms of pig lncRNA FUT3-AS1 regulating E. coli F18-bacterial diarrhea
Source: PLoS Pathog. 2022 Jun 13;18(6):e1010584. doi: 10.1371/journal.ppat.1010584 (PMC9191744; doi:10.1371/journal.ppat.1010584)
Supplement: S11 Table — K represents E. coli F18-resistant piglets; M represents E. coli F18-sensitive piglets. (DOCX) [file ppat.1010584.s023.docx]

**S11 Table. Identification of FUT3-AS1-associated proteins using RNA pull down and mass spectrometry**

| N | Unused | % Cov (95) | Accession | Name | Species | Peptides (95%) |
| --- | --- | --- | --- | --- | --- | --- |
| 1 | 42.79 | 35.87000072 | tr\|F1SGG6\|F1SGG6_PIG | KRT5 | PIG | 25 |
| 2 | 34.75 | 33.46999884 | tr\|F1S0L1\|F1S0L1_PIG | KRT14 | PIG | 21 |
| 3 | 28.92 | 28.13999951 | tr\|F1SGG3\|F1SGG3_PIG | KRT1 | PIG | 33 |
| 4 | 18.82 | 23.2099995 | tr\|I3LDS3\|I3LDS3_PIG | KRT10 | PIG | 29 |
| 5 | 12 | 8.321999758 | sp\|Q8WNW3\|PLAK_PIG | Jup | PIG | 6 |
| 6 | 10.99 | 22.40000069 | tr\|I3LDM6\|I3LDM6_PIG | KRT3 | PIG | 18 |
| 7 | 7.46 | 17.5999999 | tr\|A5A759\|A5A759_PIG | KRT2A | PIG | 13 |
| 8 | 6.3 | 22.68999964 | tr\|F1S0K1\|F1S0K1_PIG | KRT13 | PIG | 15 |
| 9 | 5.78 | 11.30999997 | tr\|F1SGI7\|F1SGI7_PIG | KRT75 | PIG | 9 |
| 10 | 5.4 | 15.44999927 | tr\|F1SRS2\|F1SRS2_PIG | LOC100302368 | PIG | 5 |
| 11 | 4.6 | 4.357000068 | tr\|I3LK01\|I3LK01_PIG | LOC100621639 | PIG | 3 |
| 12 | 3.09 | 14.55000043 | tr\|G3CKJ2\|G3CKJ2_PIG | GAPDH | PIG | 3 |
| 13 | 2.76 | 1.792999916 | tr\|F1RW75\|F1RW75_PIG | DSP | PIG | 4 |
| 14 | 2.34 | 3.245000169 | tr\|F1S073\|F1S073_PIG | ANXA2 | PIG | 1 |
| 15 | 2.03 | 1.817999966 | tr\|I3LA65\|I3LA65_PIG | DSG1 | PIG | 1 |
| 16 | 2 | 0.462599983 | tr\|I3L7I3\|I3L7I3_PIG | FLG2 | PIG | 1 |
| 17 | 2 | 3.240000084 | tr\|I3LGN8\|I3LGN8_PIG | PKP1 | PIG | 1 |
| 18 | 1.82 | 11.64999977 | sp\|P62802\|H4_PIG | Histone H4 | PIG | 1 |
| 19 | 1.71 | 5.76899983 | tr\|F2Z5U0\|F2Z5U0_PIG | RPS27A | PIG | 1 |
