# Supplementary material for: Insight into mechanisms of pig lncRNA FUT3-AS1 regulating E. coli F18-bacterial diarrhea
Source: PLoS Pathog. 2022 Jun 13;18(6):e1010584. doi: 10.1371/journal.ppat.1010584 (PMC9191744; doi:10.1371/journal.ppat.1010584)
Supplement: S12 Table — (DOCX) [file ppat.1010584.s024.docx]

**S12 Table.** **Statistics of differentially expressed miRNAs between Sutai *E. coli* F18-resistant and -sensitive piglets**

| miRNA_id | BaseMean_M | BaseMean_K | Fold Change | p-value | Sequence |
| --- | --- | --- | --- | --- | --- |
| novel_1_179916350..179916408_+_star | 14.29367941 | 131.5472195 | 0.108 | 0.032 | AACACCATTGTCACACTCCACA |
| novel_12_3405674..3405731_+_mature | 3.321704368 | 0 | Inf | 0.015 | TGGAGCCGCTTCTGAGCGCCGT |
| novel_12_46629681..46629754_+_mature | 1119.3112 | 84.41419507 | 13.259 | 0.024 | CCTGGCCTGGGAACTTCCATATGCC |
| novel_15_102286850..102286919_+_mature | 84.13631491 | 1.536967308 | 54.741 | 0.005 | CCGTGGAGAGGAACAGTTCT |
| novel_16_81524741..81524816_+_mature | 4.068508364 | 0.499458968 | 8.145 | 0.034 | GCCCCCGGTGGCGGGGGGGG |
| novel_18_20030652..20030716_+_star | 11.19724462 | 27.6535828 | 0.404 | 0.035 | TGAATTCTACCAGTGCCATACAC |
| novel_2_149522204..149522263_+_mature | 4.91812779 | 0.768483654 | 6.399 | 0.044 | TTGGCCTTCGCACCCTAACAGG |
| novel_2_84139566..84139611_+_mature | 34.50901573 | 0 | Inf | 0.001 | TGCTCTCCCGGCTCTGGCT |
| novel_3_113755731..113755774_+_mature | 255.5328306 | 60.20196593 | 4.244 | 0.034 | TGGCTGTGGCTCGACCCC |
| novel_3_17756724..17756785_+_mature | 2.148060418 | 11.01705304 | 0.194 | 0.014 | ATTCCCTTCTGGGCCTCAGGATT |
| novel_5_6244867..6244910_+_mature | 10.08670846 | 0 | Inf | 0.012 | GGCCTTTCTGTGGAGCTGG |
| novel_7_67901071..67901138_+_mature | 1.36013025 | 6.905610089 | 0.196 | 0.039 | AGTCTTGATTCTCTGTTTTGTT |
| novel_8_6216899..6216950_+_mature | 6.848115229 | 0.470518014 | 14.554 | 0.020 | GAACTGGTCTCTGGCTGCA |
| novel_9_11142542..11142607_+_mature | 13.75385923 | 0.269024686 | 51.124 | 0.006 | GTGGAGAGGAACAGTTCC |
| novel_9_72315797..72315867_+_mature | 4.669952232 | 0 | Inf | 0.004 | CGTGTCCCCTTCCCACCA |
| novel_X_56516219..56516270_+_mature | 6.777745273 | 0 | Inf | 0.041 | TCTCAGGGCTGTTGTCTG |
| novel_X_69515840..69515903_+_star | 14.66110743 | 1.536967308 | 9.538 | 0.001 | ATTCCTAGAAATTGTTCACAAT |
| ssc-miR-106a | 101.9775568 | 234.9701053 | 0.434 | 0.024 | AAAAGTGCTTACAGTGCAGGTAGC |
| ssc-miR-122-5p | 362.7510124 | 5198.390778 | 0.069 | 0.019 | TGGAGTGTGACAATGGTGTTTGT |
| ssc-miR-132 | 129.7942813 | 311.8596826 | 0.416 | 0.008 | TAACAGTCTACAGCCATGGTCG |
| ssc-miR-135 | 74.30081578 | 35.89465947 | 2.069 | 0.046 | TATGGCTTTTTATTCCTATGTGA |
| ssc-miR-1388 | 22.25843512 | 74.43657241 | 0.299 | 0.003 | AGGACTGTCAAACCTGAGAATG |
| ssc-miR-183 | 1507.31214 | 3122.134883 | 0.482 | 0.011 | TATGGCACTGGTAGAATTCACTG |
| ssc-miR-1842 | 25.55693445 | 88.37873177 | 0.289 | 0.001 | TTGGCTCTGCGAGGTCGGCT |
| ssc-miR-194b-3p | 85.61737844 | 193.704609 | 0.441 | 0.025 | CCAGTGGAGATGCTGTTACCTT |
| ssc-miR-194b-5p | 26589.1109 | 56029.3976 | 0.474 | 7.01E-06 | TGTAACAGCGACTCCATGTGGA |
| ssc-miR-20b | 163.6533353 | 404.8872812 | 0.404 | 0.012 | CAAAGTGCTCACAGTGCAGGTAG |
| ssc-miR-212 | 21.11255086 | 52.60506779 | 0.401 | 0.025 | ACCTTGGCTCTAGACTGCTTACT |
| ssc-miR-215 | 25588.03742 | 79010.34051 | 0.323 | 1.38E-05 | ATGACCTATGAATTGACAGAC |
| ssc-miR-223 | 52.45717031 | 112.7858276 | 0.465 | 0.021 | TGTCAGTTTGTCAAATACCCC |
| ssc-miR-338 | 86.06946726 | 37.75633928 | 2.279 | 0.017 | TCCAGCATCAGTGATTTTGTTG |
| novel_1_179916350..179916408_+_star | 379.0338881 | 9.81444769 | 38.619 | 0.002 | TCCCTGTCCTCCAGGAGCTC |

K represents *E. coli* F18-resistant piglets; M represents *E. coli* F18-sensitive piglets.
