# Supplementary material for: Insight into mechanisms of pig lncRNA FUT3-AS1 regulating E. coli F18-bacterial diarrhea
Source: PLoS Pathog. 2022 Jun 13;18(6):e1010584. doi: 10.1371/journal.ppat.1010584 (PMC9191744; doi:10.1371/journal.ppat.1010584)
Supplement: S13 Table — (DOCX) [file ppat.1010584.s025.docx]

**S13 Table. ceRNA construction of FUT3-AS1 (TCONS_00183659)-miRNAs-*FUT3***

| miRNA | Target | Total Score | miRNA Length | Target Length | Positions |
| --- | --- | --- | --- | --- | --- |
| novel_1_179916350..179916408_+_star | TCONS_00183659 | 315 | 22 | 5833 | 5353, 5090 |
| novel_12_3405674..3405731_+_mature | TCONS_00183659 | 285 | 22 | 5833 | 5758, 1057 |
| novel_12_46629681..46629754_+_mature | TCONS_00183659 | 1144 | 25 | 5833 | 1856, 2002, 5104, 1400, 5464, 1017, 3475 |
| novel_15_102286850..102286919_+_mature | TCONS_00183659 | 284 | 20 | 5833 | 4676, 654 |
| novel_16_81524741..81524816_-_mature | TCONS_00183659 | 282 | 20 | 5833 | 17, 238 |
| novel_16_81524741..81524816_-_mature | *FUT3* | 142 | 20 | 1565 | 62 |
| novel_18_20030652..20030716_-_star | *FUT3* | 156 | 23 | 1565 | 872 |
| novel_18_20030652..20030716_-_star | TCONS_00183659 | 142 | 23 | 5833 | 3428 |
| novel_2_149522204..149522263_-_mature | TCONS_00183659 | 148 | 22 | 5833 | 42 |
| novel_2_149522204..149522263_-_mature | *FUT3* | 148 | 22 | 1565 | 87 |
| novel_3_113755731..113755774_-_mature | TCONS_00183659 | 603 | 18 | 5833 | 1046, 297, 3274, 2055 |
| novel_3_113755731..113755774_-_mature | *FUT3* | 140 | 18 | 1565 | 654 |
| novel_3_17756724..17756785_+_mature | TCONS_00183659 | 893 | 23 | 5833 | 88, 5117, 309, 2370, 19, 1027 |
| novel_3_17756724..17756785_+_mature | *FUT3* | 140 | 23 | 1565 | 64 |
| novel_5_6244867..6244910_-_mature | TCONS_00183659 | 301 | 19 | 5833 | 580, 2376 |
| novel_7_67901071..67901138_+_mature | TCONS_00183659 | 144 | 22 | 5833 | 3605 |
| novel_9_11142542..11142607_+_mature | TCONS_00183659 | 144 | 18 | 5833 | 4331 |
| novel_9_72315797..72315867_-_mature | TCONS_00183659 | 146 | 18 | 5833 | 4922 |
| novel_X_56516219..56516270_+_mature | TCONS_00183659 | 458 | 18 | 5833 | 1051, 5260, 3582 |
| novel_X_69515840..69515903_+_star | TCONS_00183659 | 445 | 22 | 5833 | 4597, 513, 5398 |
| ssc-miR-106a | TCONS_00183659 | 290 | 24 | 5833 | 4576, 3988 |
| ssc-miR-132 | *FUT3* | 140 | 22 | 1565 | 185 |
| ssc-miR-135 | TCONS_00183659 | 142 | 23 | 5833 | 748 |
| ssc-miR-194b-3p | TCONS_00183659 | 153 | 22 | 5833 | 3338 |
| ssc-miR-194b-5p | TCONS_00183659 | 157 | 22 | 5833 | 956 |
| ssc-miR-20b | TCONS_00183659 | 150 | 23 | 5833 | 4577 |
| ssc-miR-212 | *FUT3* | 159 | 23 | 1565 | 85 |
| ssc-miR-212 | TCONS_00183659 | 159 | 23 | 5833 | 40 |
| ssc-miR-215 | *FUT3* | 140 | 21 | 1565 | 1410 |
| ssc-miR-338 | TCONS_00183659 | 149 | 22 | 5833 | 5145 |
| ssc-miR-4334-3p | TCONS_00183659 | 439 | 20 | 5833 | 3124, 242, 4125 |
