# Supplementary material for: Insight into mechanisms of pig lncRNA FUT3-AS1 regulating E. coli F18-bacterial diarrhea
Source: PLoS Pathog. 2022 Jun 13;18(6):e1010584. doi: 10.1371/journal.ppat.1010584 (PMC9191744; doi:10.1371/journal.ppat.1010584)
Supplement: S14 Table — (DOCX) [file ppat.1010584.s026.docx]

**S14 Table. Key differentially abundant proteins between the pig siFUT3-AS1 group and the control group IPEC-J2 cells based on iTRAQ proteome analysis.**

| Accession | Species/Protein name | Protein length | siFUT3-AS1: control | p-value |
| --- | --- | --- | --- | --- |
| tr\|Q70D54\|Q70D54_PIG | OS=Sus scrofa GN=ST3GAL3 PE=3 SV=1 | 374 | 0.437846 | 0.045642 |
| sp\|Q864U6\|B3GL1_PIG | OS=Sus scrofa GN=B3GALNT1 PE=1 SV=1 | 331 | 0.428966 | 0.0371 |
| sp\|Q10982\|FUT2_PIG | OS=Sus scrofa GN=FUT2 PE=1 SV=1 | 340 | 0.291716 | 0.011469 |
